# Genome Diversification Mechanism of Rodent and Lagomorpha Chemokine Genes

**DOI:** 10.1155/2013/856265

**Published:** 2013-08-07

**Authors:** Kanako Shibata, Hisayuki Nomiyama, Osamu Yoshie, Sumio Tanase

**Affiliations:** ^1^School of Health Sciences, Kumamoto University, Kuhonji, Kumamoto 860-0976, Japan; ^2^Department of Molecular Enzymology, Kumamoto University, Graduate School of Medical Sciences, Honjo, Kumamoto 860-8556, Japan; ^3^Department of Microbiology, Kinki University, Faculty of Medicine, Osaka-Sayama, Osaka 589-8511, Japan; ^4^Department of Biomedical Laboratory Sciences, Faculty of Life Sciences, Kumamoto University, Kuhonji, Kumamoto 860-0976, Japan

## Abstract

Chemokines are a large family of small cytokines that are involved in host defence and body homeostasis through recruitment of cells expressing their receptors. Their genes are known to undergo rapid evolution. Therefore, the number and content of chemokine genes can be quite diverse among the different species, making the orthologous relationships often ambiguous even between closely related species. Given that rodents and rabbit are useful experimental models in medicine and drug development, we have deduced the chemokine genes from the genome sequences of several rodent species and rabbit and compared them with those of human and mouse to determine the orthologous relationships. The interspecies differences should be taken into consideration when experimental results from animal models are extrapolated into humans. The chemokine gene lists and their orthologous relationships presented here will be useful for studies using these animal models. Our analysis also enables us to reconstruct possible gene duplication processes that generated the different sets of chemokine genes in these species.

## 1. Introduction

Chemokines are a family of small cytokines whose major tasks are tissue recruitment of leukocytes and lymphocytes under inflammatory and homeostatic conditions [1–3]. They are also involved in angiogenesis, organogenesis, tumor metastasis, and viral infection. Chemokines can be divided into five subfamilies, CXC, CC, XC, CX3C, and CX, based on the arrangement of the two N-terminal conserved cysteine residues [4]. The CX subfamily has been found only in zebrafish so far, while other subfamilies are present in vertebrates [5, 6]. There are two large clusters of chemokine genes, one consisting of CXC and the other CC chemokines, in mammalian genomes. The human CXC and CC chemokine major clusters are located on chromosomes 4 and 17, respectively [7]. CXC and CC chemokine genes are also found in several miniclusters or as a single-gene in the genome. Chemokines can be also classified based on their mode of expression and function [1, 7, 8]. Inflammatory chemokines such as CXCL8 (also known as IL-8) are upregulated under conditions of inflammation, while homeostatic chemokines such as CXCL12 (also known as SDF-1) are produced constitutively at noninflamed sites, controlling from cell trafficking in the embryo to leukocyte homing for immune surveillance. The genes for inflammatory chemokines are mostly located in the major clusters, while the genes for homeostatic chemokines are usually located in other chromosomal sites. Some chemokines have both functions and thus are called dual-function chemokines [7, 8].

There are at least 44 and 46 chemokine genes in the human and mouse genomes, respectively [4]. Chemokine genes, particularly those in the major clusters, evolved rapidly at a rate much faster than other genes involved in host defence through recurrent gene duplication and deletion events during mammalian evolution. Such events occurred even after the diversification of mammalian species, generating species-specific chemokine genes [7]. Furthermore, chemokines duplicated relatively recently, most of which are inflammatory chemokines, tend to be promiscuous in ligand-receptor interactions: a single-chemokine receptor responds to multiple chemokines and, conversely, one chemokine acts on several chemokine receptors [9]. Therefore, there could be ambiguity in the orthologous relationships between the chemokine genes of different species. In contrast, one homeostatic chemokine generally recognizes only one chemokine receptor. For these chemokine genes, one-to-one orthologous relationships are prevalent. In addition, humans and mouse strains have copy number variations in some chemokine genes [10, 11]. The copy numbers of tandemly duplicated CCL3- and CCL4-like genes vary among human individuals and have been implicated in the susceptibility and disease progression of HIV infection [10, 12–14].

**Figure 1:**
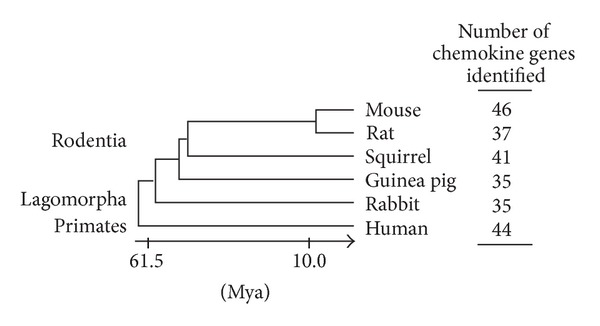
Number of chemokine genes identified in the genomes of rodents and rabbit. Phylogenetic relationships of rodents, rabbit, and human are also shown. Divergence times (Mya, million years ago) [16] are not to scale. Sequences of the chemokines used for analyses in this study and the phylogenetic tree are shown in the Supplementary Material available online at http://dx.doi.org/10.1155/2013/856265.

Rodents and rabbit provide valuable animal models for experimental and toxicological studies and drug development, and their genomes have been completely sequenced or are being sequenced. When interpreting data from experiments using these animals, however, it may be important to know the content of chemokine genes in each species and their orthologous relationships to human counterparts. Previously, we have identified chemokine and chemokine receptor genes in many vertebrate species and revealed their evolutionary processes. However, we have included only one or two rodent species in those analyses [4, 7, 15]. Here, we compare the content and organization of chemokine genes in Rodentia (mouse, rat, squirrel, and guinea pig) and Lagomorpha (rabbit) by analyzing their genome sequences (see Figure 1 for phylogenetic relationships of these animals). Lagomorpha and Rodentia are grouped in the Glires superorder, which is most closely related to primates. The comparison revealed species-specific chemokine genes and also birth-and-death processes of the chemokine genes during evolution of Glires.

## 2. The CXC Major Cluster

**Figure 2:**
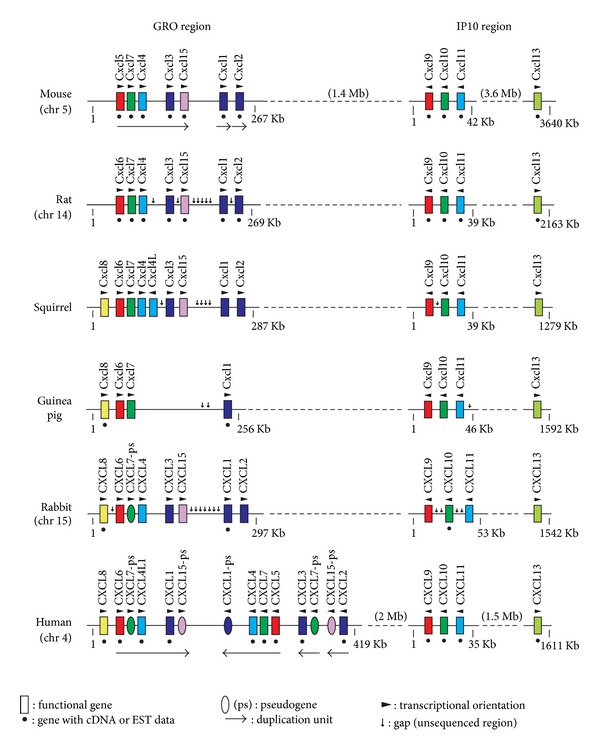
Genomic organization of the CXC chemokine major cluster. The maps shown are based on the Ensembl Genome Browser and our analyses. The gene symbols for the two human CXCL7 pseudogenes (CXCL7-ps pseudogenes between CXCL6 and CXCL4L1 and between CXCL3 and CXCL15-ps) and one human CXCL1 pseudogene (CXCL1-ps) are PPBPP1 [18], PPBPP2, and CXCL1P [19], respectively. Genome sequences used for analysis with BLAST search are GRCm38 (mouse, *Mus musculus*), Rnor_5.0 (rat, *Rattus norvegicus*), spetri2 (squirrel, *Ictidomys tridecemlineatus*), cavPor3 (guinea pig, *Cavia porcellus*), oryCun2 (rabbit, *Oryctolagus cuniculus*), and GRCh37 (human, *Homo sapiens*).

Previously, we have proposed to subdivide the CXC major cluster into two separate regions, GRO and IP10, which are located 2 Mb apart on the human chromosome 4 [17] (Figure 2). The human GRO region contains 9 genes (CXCL8, CXCL6, CXCL4L1, CXCL1, CXCL4, CXCL7, CXCL5, CXCL3, and CXCL2), while the mouse GRO region is located on chromosome 5 and contains 7 genes (Cxcl5, Cxcl7, Cxcl4, Cxcl3, Cxcl15, Cxcl1, and Cxcl2) (Figure 2). The name of the region “GRO” was taken from the chemokines GRO1, GRO2, and GRO3 (their systematic names are CXCL1, CXCL2, and CXCL3) in the region, while the name of the “IP10” was derived from a representative chemokine IP-10 (CXCl10). The chemokine names used throughout this paper are based on the proposed systematic nomenclature for the chemokine family [2, 3]. This nomenclature system differs in some chemokine names from the official human and mouse gene symbols [3, 7]. For example, instead of CXCL4 (Cxcl4), PF4 (human) and Pf4 (rodents) are still used for gene symbols (HUGO Gene Nomenclature Committee, http://www.genenames.org/; Mouse Genomic Nomenclature Committee, http://www.informatics.jax.org/mgihome/nomen/). Furthermore, as described later, there are some discrepancies between the human and mouse gene symbols. In this paper, we conform the names of chemokine genes identified in the genomes of squirrel, guinea pig, and rabbit to the nomenclature of mouse chemokines in order to avoid confusion. 

The gene organizations of the GRO regions of rat, squirrel, guinea pig, and rabbit are quite similar to that of mouse GRO region (Figure 2). There are, however, minor differences in each species. It is widely known that Cxcl8 gene is absent from the mouse and rat [20, 21]. However, other rodents and rabbit contain CXCL8 gene, suggesting that Cxcl8 gene was deleted in the lineage of muroid rodents. However, mouse gene symbol for Cxcl15 has recently been changed to Il-8 (synonymous with Cxcl8). This new symbol might be erroneously assigned because mouse Cxcl15 exhibits a low similarity (31%) to human CXCL8, and some species (squirrel and rabbit) contain both genes (Figure 2). Even human contains a pseudogene for CXCL15 (our unpublished result). Among the animals investigated, guinea pig has less GRO chemokines than other animals. The animal lacks Cxcl4, Cxcl3, Cxcl15, and Cxcl2 genes. Since the genome sequencing of guinea pig is still in progress, some of the genes may be present in the gaps still not covered by genome sequencing.

**Figure 3:**
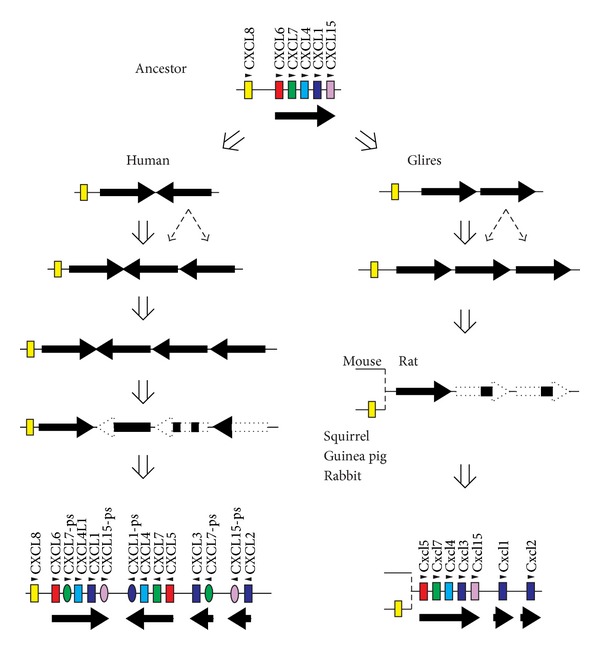
Proposed diversification mechanism of GRO chemokines. Genome sequences were analyzed with PipMaker dot plots (http://pipmaker.bx.psu.edu/pipmaker/).

Human GRO chemokine genes have expanded more extensively than those of rodents and rabbit due to lineage-specific duplication events.  Figure 3 shows the predicted duplication events including 5 chemokine genes (CXCL6, CXCL7, CXCL4, CXCL1, and CXCL15) in each lineage. The region encompassing the 5 chemokine genes might have existed in the ancestor genome common to human, rodents, and rabbit and might have been served as a duplication unit. In the human lineage, the region was first inversely duplicated. Then, the one unit was tandemly duplicated twice, resulting in the generation of three tandem copies. Differential rearrangements such as gene deletion and inactivation in each duplicated region during the evolution have led to the present organization. On the other hand, in the lineages of rodents and rabbit, the ancestral 5-gene region was first tandemly duplicated and then the one unit was tandemly duplicated again. Then, extensive gene deletions might have occurred in the duplicated regions. Obviously, such lineage-specific duplications and rearrangements can cause confusions in orthologue assignment. However, considering the duplication processes in each lineage, it is now possible to deduce the true orthologous relationships of these genes. Without such information, it was impossible to determine the orthologs of GRO genes because, in general, paralogous GRO chemokines in one species are more closely related to each other than to GRO chemokines of other species [20]. Thus, it is apparent that human CXCL1 and CXCL6 correspond to mouse Cxcl3 and Cxcl5, respectively [7]. Similarly, human CXCL2 or CXCL3 should be orthologous to mouse Cxcl1 and Cxcl2.

Recently, it has been shown that circulating CXCL5 is highly increased during obesity in mice and that CXCL5 can induce insulin resistance [22]. Furthermore, the authors showed that CXCL5 serum concentration is increased in obese patients with insulin resistance, suggesting that CXCL5 also promotes insulin resistance in humans. However, CXCL5 in serum of human patients is not dramatically increased compared to those of mice in obese state. Since the human counterpart of mouse Cxcl5 is CXCL6 from a functional [23, 24] and genomical point of view, a serum level of CXCL6 in patients should be examined.

The DNA rearrangement in the GRO regions occurred relatively recently. Most of the GRO genes are inflammatory chemokines and one of the duplicated copies might have been mutated in order to counteract the molecular mimicry by viruses and maintain the diversity of host defense proteins [25–27]. 

In contrast, the IP10 regions of rodents and rabbit are quite similar to that of human (Figure 2). IP10 genes are dual-function or homeostatic chemokines and are older than the GRO chemokines. Because of the importance of their physiological functions, they may be conserved during evolution.

## 3. The CC Major Cluster

**Figure 4:**
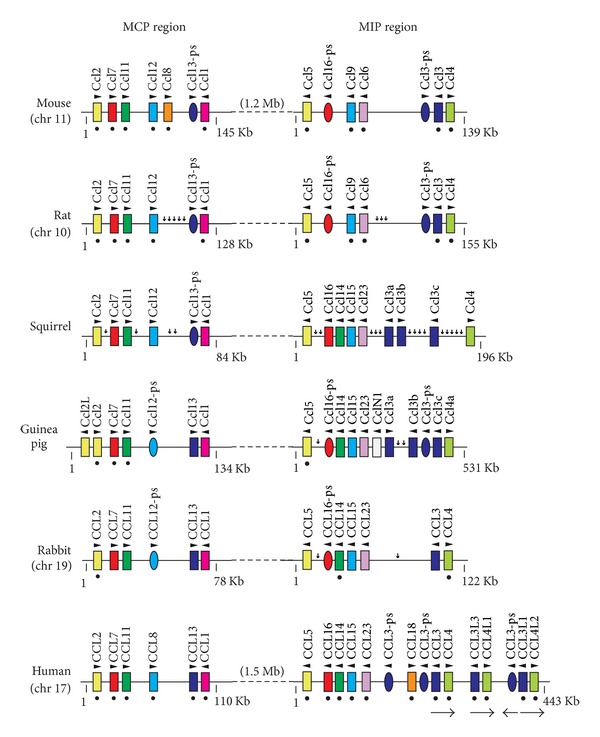
Genomic organization of the CC chemokine major cluster. The maps shown are based on the Ensembl Genome Browser and our analyses. Although the human gene names for CCL3-like and CCL4-like genes shown in the map are based on the gene assignments of the Hugo Gene Nomenclature Committee, Colobran et al. [10, 29] proposed different names for these genes based on their coding sequence similarity. Human CCL3P1 (previous gene symbol CCL3L2 [30]) located between CCL4L1 and CCL3L1 is indicated as CCL3-ps for simplicity. Two human CCL3-ps genes on each side of CCL18 were identified in our previous study [31]. The gene symbol for the mouse Ccl16 pseudogene is Ccl16-ps [32].

We have also proposed to subdivide the CC major cluster into the MCP and MIP regions, which are located 1.5 Mb apart on the human chromosome 17 [17] (Figure 4). The MIP region can be further divided into two groups [28]. 

The MCP region is relatively well conserved among the rodent, rabbit, and human genomes with only some minor species-specific gene changes. For example, human and mouse contain the same number of functional genes in the MCP region (human, CCL2, CCL7, CCL11, CCL8, CCL13, and CCL1; mouse, Ccl2, Ccl7, Ccl11, Ccl12, Ccl8, and Ccl1), but each has one species-specific gene (human CCL13; mouse Ccl8). The name of the region “MCP” was taken from the chemokines MCP-1 (CCL2), MCP-2 (CCL8), MCP-3 (CCL7), and MCP-4 (CCL13). Although CCL8 gene is present in human genome, the analysis of the receptor usage has recently revealed that its true mouse ortholog is Ccl12 [33]. Our previous ortholog assignment based on the genomic analysis also supports this biological evidence [31]. CCL13 gene is present in guinea pig and rabbit, but this gene is inactivated in mouse, rat, and squirrel. Guinea pig has an additional Ccl2-like gene located next to Ccl2 gene. Guinea pig and rabbit have a pseudogene for CCL8, while mouse, rat, and squirrel have CCL13-ps.

**Figure 5:**
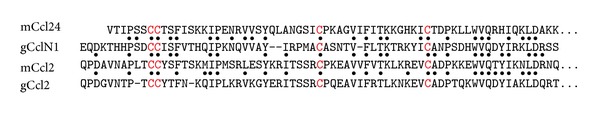
A novel guinea pig chemokine CclN1. Amino acid sequence of mature guinea pig CclN1 is aligned with those of mouse Ccl2, Ccl24, and guinea pig Ccl2. Red letters indicate the cysteine residues conserved among chemokines. The C-terminal regions of mouse Ccl2, Ccl24, and guinea pig Ccl2 are omitted. g, guinea pig; m, mouse.

The MIP region is quite diversified among these species. The mouse and human MIP regions contain 5 and at least 8 functional genes, respectively (mouse, Ccl5, Ccl9, Ccl6, Ccl3, and Ccl4; human, CCL5, CCL16, CCL14, CCL15, CCL23, CCL18, CCL3, and CCL4) (Figure 4). The name of the region “MIP” was taken from the chemokines MIP-1*α* (CCL3) and MIP-1*β* (CCL4). These closely related genes, CCL3 and CCL4, are arranged side-by-side and constitute a duplication unit in the human genome. The copy numbers of the duplicated CCL3- and CCL4-like genes such as CCL3L1 and CCL4L1 are now known to be variable in each individual [10, 34] although we and others found the copy variations of the genes more than 20 years ago [30, 35, 36]. The copy number variation of the duplication unit (ca. 100 kb) has been proposed to underlie susceptibility to HIV. In contrast, rodents and rabbit have only one pair of CCL3 and CCL4. However, CCL3 gene has independently been duplicated during evolution, and most species including mouse and human have one or more copies of functional or inactivated CCL3 genes. In the human genome, CCL18 gene may have been generated by fusion of two such duplicated CCL3-like genes with selective usage of some exons [37, 38]. Besides CCL3 and CCL4 genes, there are other differences in the MIP region. Contrary to the human CCL16, mouse Ccl16 is inactivated [32], and the CCL16 genes of the other rodents and rabbit are also apparently pseudogenes. One exception is squirrel CCL16, which seems to be functional. Furthermore, mouse and rat lack CCL14 gene. Interestingly, guinea pig contains a novel chemokine gene (termed CclN1) between Ccl23 and Ccl3a genes. The chemokines that show high similarity to CclN1 are mouse Ccl24 (42%), mouse Ccl2 (38%), and guinea pig Ccl2 (41%) (Figure 5). Although there is no evidence that guinea pig CclN1 is expressed, it might be a substitute for Ccl24 since guinea pig Ccl24 is a pseudogene (see next). As we have previously described, mouse and rat Ccl9 and Ccl6 genes are in fact orthologous to human Ccl15 and CCL23 genes, respectively [31]. 

## 4. Other Chemokines

Most chemokines isolated or purified until nearly two decades ago were cluster-forming, inflammatory chemokines. One of a few exceptions was CXCL12 now categorized as a homeostatic chemokine, whose gene is located on a chromosome different from the other chemokine genes. This fact suggested that noncluster chemokines might have physiological roles different from inflammatory chemokines and led to the identification of a number of homeostatic chemokines [39, 40]. 

**Figure 6:**
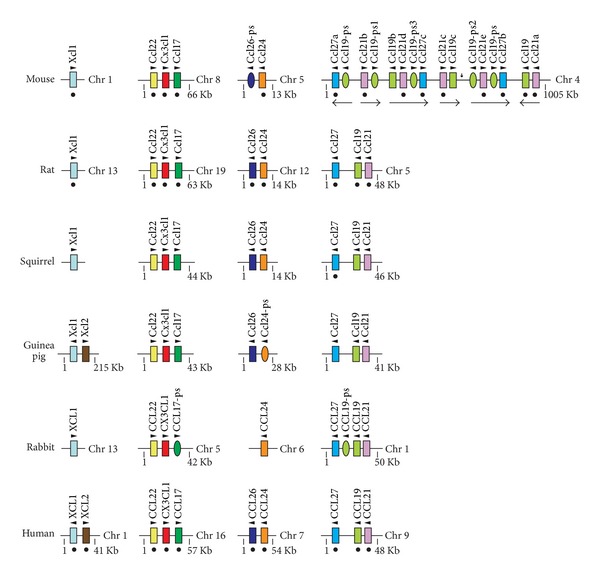
Genomic organization of chemokine miniclusters. Four miniclusters locating on different chromosomes are shown.

Noncluster chemokines can be classified into miniclusters and single genes located on different chromosomes in the human genome (Figure 6). One of miniclusters is XCL1-XCL2 in the human genome. Like human, guinea pig has two XC chemokine genes, Xcl1 and Xcl2, whereas other rodents and rabbit have only one Xcl1 gene. All species have the CCL22-CX3CL1-CCL17 minicluster. Although CCL17 gene is a pseudogene in rabbit, CCL22 may replace the chemokine in rabbit because both bind CCR4. Another minicluster consists of CCL26 and CCL24 (Figure 6). Mouse Ccl26 may be a pseudogene since no cDNA or EST has been reported [41], whereas two EST clones have been isolated for rat Ccl26 (UniGene ID: 1532479). Guinea pig Ccl24 gene is apparently a pseudogene due to base changes. Rabbit may lack Ccl26 gene. Since CCL24 and CCl26 are among the multiple CCR3 ligands, lack of one of the gene products may not cause serious physiological problems in those species. 

It has been shown that CCL26 is the most overexpressed in patients with eosinophilic esophagitis (EE), a disease characterized by the accumulation of eosinophils in the esophagus and that CCL26 has a crucial role in eosinophil recruitment in EE [42]. However, Ccl26 is likely a pseudogene in mice. Although knockout mice lacking Ccr3, which binds chemokines Ccl26 and other several eosinophil chemoattractants, do not develop experimental EE, it is still not known which chemokine(s) is responsible for the eosinophil recruitment in the EE model in mice.

**Figure 7:**
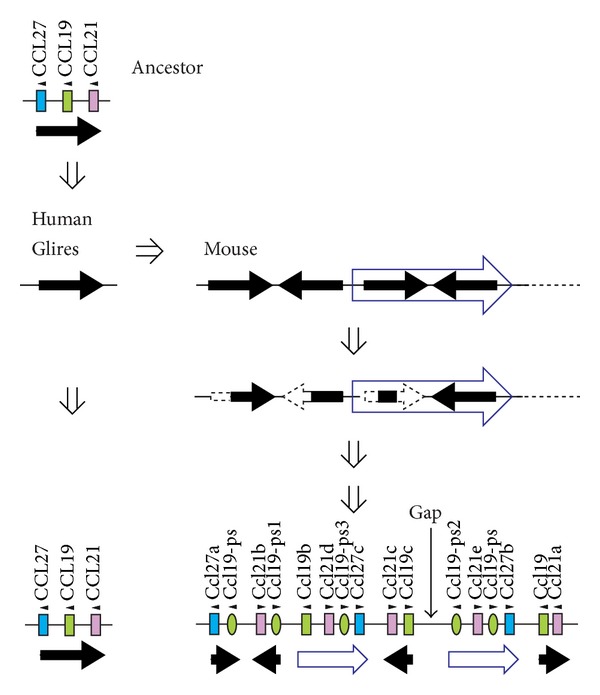
Proposed diversification mechanism of mouse Ccl27-Ccl19-Ccl27 minicluster chemokine genes.

Another minicluster is CCL27-CCL19-CCL21 (Figure 6). In the mouse genome, however, the cluster has considerably expanded [11]. As shown in Figure 7, the ancestral 3-gene segment might have been first inversely duplicated and then the whole inverse repeats might have been duplicated again. After these duplications, some genes in each segment might have been lost or inactivated during evolution. Thus, the C57BL/6J minicluster includes 3 Ccl27, 3 Ccl19, and 5 Ccl21 functional genes and 5 Ccl19 pseudogenes. However, the sequence still contains a gap and more chemokine genes may be identified within the cluster.

Seven chemokine genes (CXCL12, CXCL14, CXCl16, CXCl17, CCL20, CCL25, and CCL28) are singly located in the human and mouse genomes. These genes are homeostatic or dual-function chemokines. Other rodents have the same 7 genes. Although rabbit CXCL12 and CXCL14 genes have not yet been identified, they may be found in the gaps, given that their lack should have a detrimental effect. For example, mice lacking CXCL12 die perinatally [43].

## 5. Conclusions

Mammalian species have considerable differences in the number and the content of chemokine genes [4, 7, 44, 45]. The differences are mainly caused by the expansion of the major CXC and CC chemokine gene clusters that mostly encode inflammatory chemokines. Such differences are also seen among rodents and rabbit (Figures 2 and 4). It seems likely that recurrent segmental duplication events mainly increased the chemokine gene numbers (Figures 3 and 7). Single-gene duplications, gene inactivations, and deletions of small segments might have followed in species-specific manner. Although inactivation and deletion of duplicated genes decrease the total number of genes, such events also contributed a great deal to the generation of lineage- and species-specific genes. For example, CXCL8 gene is missing only in the lineage of mouse and rat, while Ccl13 gene was inactivated in the lineage of mouse, rat, and squirrel. In humans and mice, increases in chemokine genes may still be ongoing in the gene clusters since the numbers of some chemokine genes are variable in human individuals and in mouse strains (Figures 4 and 6). These DNA rearrangements such as duplication, deletion, and inactivation have generated the species diversity of the chemokine system.

Chemokines generated by recent gene duplications tend to show high similarity to each other. Gene conversion [46] between the nearest neighbors may also contribute to maintaining the high similarity. The determination of orthologs based solely on the sequence similarity is therefore difficult and may have caused some confusions in the chemokine terminology. Specifically, incorrect annotation of mouse chemokine genes might have caused considerable confusion in extrapolating mouse data to humans. The genome maps that show the orthologous relationships of various chemokine genes (Figures 2, 4, and 6) will therefore be of great help in research using rodents and rabbit.

In contrast to the chemokine ligands, chemokine receptors are more conserved among mammals [15]. There are 24 and 25 chemokine receptor genes so far identified in the human and mouse genomes, respectively [15, 47, 48]. They are well conserved between these two species. Similarly, the chemokine receptors of other rodents and rabbit (not shown) are relatively well conserved although there are some differences among the species. Like mouse, rat and guinea pig have Ccr1-like1, which is highly similar to Ccr1, but squirrel and rabbit lack this gene as human. In addition, rabbit CXCR1 and CXCR2 genes appear to be inactivated due to frameshifts. However, it is unlikely that they are both pseudogenes. Given that human CXCL6 and CXCL8 bind only to CXCR1 and CXCR2 and rabbit has both genes, at least one of the receptors should be functional. Therefore, there may be some sequence editing errors in the rabbit CXCR1 and/or CXCR2 gene. In fact, rabbit cDNAs for both genes were isolated. This example indicates that the identification of a ligand gene in one genome and its orthologous assignment to human or mouse gene is useful in identification and annotation of its receptor gene.

## Supplementary Material

Amino acid sequences of the rodent and rabbit chemokines together with the phylogenetic tree.
