# Supplementary material for: Genome Diversification Mechanism of Rodent and Lagomorpha Chemokine Genes
Source: Biomed Res Int. 2013 Aug 7;2013:856265. doi: 10.1155/2013/856265 (PMC3749542; doi:10.1155/2013/856265)
Supplement: Supplementary file 1 — Amino acid sequences of the rodent and rabbit chemokines together with the phylogenetic tree. [file 856265.f1.pdf]

has, human
mmu, mouse
rno, rat
ocu, rabbit
cpo, guinea pig
itr, squirrel

>hsaCXCL8 ENSG00000169429 chr4
mtsklavallaaflisaalcegAVLPRSAKELRCQCIKTYSKPFHPKFIKELRVIESGPHCANTEIIVKL
SDGRELCLDPKENWVQRVVEKFLKRAENS

>hsaCXCL6 ENSG00000124875 chr4
mslpssraarvpgpsgslcallalllllltppgplasAGPVSAVLTELRCTCLRVTLRVNPKTIGKLQVFP
AGPQCSKVEVVASLKNGKQVCLDPEAPFLKKVIQKILDSGNKKN

>hsaCXCL4L1 ENSG00000109272 chr4
mssaarsrltratrqemlflalllllpvvvafaraEAEEDGDLQCLCVKTTSQVRPRHITSLEVIKAGPHC
PTAQLIATLKNGRKICLDLQALLYKKIIKEHLES

>hsaCXCL1 ENSG00000163739 chr4
maraalsaapsnprllrvalllllllvaagrraagASVATELRCQCLQTLQGIHPKNIQSVNVKSPGPHCA
QTEVIATLKNGRKACLNPASPIVKKIIEKMLNSDKSN

>hsaCXCL4 ENSG00000163737 chr4
mssaagfcasrpgllflgllllplvvafasaEAEEDGDLQCLCVKTTSQVRPRHITSLEVIKAGPHCPTA
QLIATLKNGRKICLDLQAPLYKKIIKKLLES

>hsaCXCL7 ENSG00000163736 chr4
mslrldttpscnsarplhalqvllllsllltalaSSTKGQTKRNLAKGKEESLDSDLYAELRCMCIKTTS
GIHPKNIQSLEVIGKGTHCNQVEVIATLKDGRKICLDPDAPRIKKIVQKKLAGDESAD

>hsaCXCL5 ENSG00000163735 chr4
msllssraarvpgpssslcallvlllllltqpgpiasAGPAAAVLRELRCVCLQTTQGVHPKMISNLQVFA
IGPQCSKVEVVASLKNGKEICLDPEAPFLKKVIQKILDGGNKEN

>hsaCXCL3 ENSG00000163734 chr4
mahatlsaapsnprllrvalllllllvaasrraagASVVTELRCQCLQTLQGIHLKNIQSVNVRSPGPHCA
QTEVIATLKNGKKACLNPASPMVQKIIEKILNKGSTN

>hsaCXCL2 ENSG00000081041 chr4
maratlsaapsnprllrvalllllllvaasrraagAPLATELRCQCLQTLQGIHLKNIQSVKVKSPGPHCA
QTEVIATLKNGQKACLNPASPMVKKIIEKMLKNGKSN

>hsaCXCL9 ENSG00000138755 chr4
mkksgvlfllgiillvligvqgTPVVRKGRCSCISTNQGTIHLQSLKDLKQFAPSPSCEKIEIIATLKNG
VQTCLNPDSADVKELIKKWEKQVSQKKKQKNGKKHQKKKVLKVRKSQRSRQKKTT

>hsaCXCL10 ENSG00000169245 chr4
mnqtailiccliflltlsgiqgVPLSRTVRCTCISISNQPVNPRSLEKLEIIPASQFCPRVEIIATMKKKG
EKRCLNPESKAIKNLLKAVSKERSKRSP

>hsaCXCL11 ENSG00000169248 chr4
msvkgmaialavilcatvvqgFPMFKRGRCLCIGPGVKAVKVADIEKASIMYPSNNCDKIEVIITLKENK
GQRCLNPKSKQARLIIKKVERKNF

>hsaCXCL13 ENSG00000156234 chr4

mkfistslllmllvsslspvqgVLEVYYTSLRCRCVQESSVFIPRRFIDRIQILPRGNGCPRKEIIVWKK
NKSIVCVDPQAEWIQRMMEVLRKRSSSTLPVPVFKRKIP

>hsaCXCL12 ENSG00000107562 chr10
mnakvvvvlvlvltalclsdgKPVSLSYRCPCRFFESHVARANVKHLKILNTPNCALQIVARLKNNNRQV
CIDPKLKWIQEYLEKALNKRFKM

>hsaCXCL14 ENSG00000145824 chr5
msllprrappvsmrllaaallllllalytarvdgSKCKCSRKGPKIRYSDVKKLEMKPKYPHCEEKMVII
TTKSVSRYRGQEHCLHPKLQSTKRFIKWYNAWNEKRRVYEE

>hsaCXCL16 ENSG00000161921 chr17
msgsqsevapspqsprspemgrdlrpgsrvlllllllllvyltqpgngNEGSVTGSCYCGKRISSDSPPS
VQFMNRLRKHLRAYHRCLYYTRFQLLSWSVCGGNKDPWVQELMSCLDLKECGHAYSGIVAHQKHLLPTSP
PISQASEGASSDIHTPAQMLLSTLQSTQRPTLPVGSLSSDKELTRPNETTIHTAGHSLAAGPEAGENQKQ
PEKNAGPTARTSATVPVLCLLAIIFILTAALSYVLCKRRRGQSPQSSPDLPVHYIPVAPDSNT

>hsaCXCL17 ENSG00000189377 chr19
mkvlisslllllplmlmsmvssSLNPGVARGHRDRGQASRRWLQEGGQECECKDWFLRAPRRKFMTVSGL
PKKQCPCDHFKGNVKKTRHQRHHRKPNKHSRACQQFLKQCQLRSFALPL

>hsaCCL2 ENSG00000108691 Chr17
mkvsaallcllliaatfipqglaQPDAINAPVTCCYNFTNRKISVQRLASYRRITSSKCPKEAVIFKTIV
AKEICADPKQKWVQDSMDHLDKQTQTPKT

>hsaCCL7 ENSG00000108688 Chr17
mkasaallcllltaaafspqglaQPVGINTSTTCCYRFINKKIPKQRLESYRRTTSSHCPREAVIFKTKL
DKEICADPTQKWVQDFMKHLDKKTQTPKL

>hsaCCL11 ENSG00000172156 Chr17
mkvsaallwllliaaafspqglaGPASVPTTCCFNLANRKIPLQRLESYRRITSGKCPQKAVIFKTKLAK
DICADPKKKWVQDSMKYLDQKSPTPKP

>hsaCCL8 ENSG00000108700 Chr17
mkvsaallclllmaatfspqglaQPDSVSIPITCCFNVINRKIPIQRLESYTRITNIQCPKEAVIFKTKR
GKEVCADPKERWVRDSMKHLDQIFQNLKP

>hsaCCL13 ENSG00000181374 Chr17
mkvsavllclllmtaafnpqglaQPDALNVPSTCCFTFSSKKISLQRLKSYVITTSRCPQKAVIFRTKLG
KEICADPKEKWVQNYMKHLGRKAHTLKT

>hsaCCL1 ENSG00000108702 Chr17
mqiittalvclllagmwpedvdsKSMQVPFSRCCFSFAEQEIPLRAILCYRNTSSICSNEGLIFKLKRGK
EACALDTVGWVQRHRKMLRHCPSKRK

>hsaCCL5 ENSG00000161570 chr17
mkvsaaalaviliatalcapasaSPYSSDTTPCCFAYIARPLPRAHIKEYFYTSGKCSNPAVVFVTRKNR
QVCANPEKKWVREYINSLEMS

>hsaCCL16 ENSG00000161573 chr17
mkvseaalsllvliliitsasrsQPKVPEWVNTPSTCCLKYYEKVLPRRLVVGYRKALNCHLPAIIFVTK
RNREVCTNPNDDWVQEYIKDPNLPLLPTRNLSTVKIITAKNGQPQLLNSQ

>hsaCCL14 ENSG00000213494 chr17
mkisvaaipffllitialgTKTESSSRGPYHPSECCFTYTTYKIPRQRIMDYYETNSQCSKPGIVFITKR
GHSVCTNPSDKWVQDYIKDMKEN

>hsaCCL15 ENSG00000161574 chr17
mkvsvaalsclmlvavlgsqaQFINDAETELMMSKLPLENPVVLNSFHFAADCCTSYISQSIPCSLMKSY

FETSSECSKPGVIFLTKKGRQVCAKPSGPGVQDCMKKLKPYSI

>hsaCCL23 ENSG00000167236 chr17
mkvsvaalsclmlvtalgsqaRVTKDAETEFMMSKLPLENPVLLDMLWRRKIGPQMTLSHAAGFHATSAD
CCISYTPRSIPCSLLESYFETNSECSKPGVIFLTKKGRRFCANPSDKQVQVCVRMLKLDTRIKTRKN

>hsaCCL18 ENSG00000006074 chr17
mkglaaallvlvctmalcscAQVGTNKELCCLVYTSWQIPQKFIVDYSETSPQCPKPGVILLTKRGRQIC
ADPNKKWVQKYISDLKLNA

>hsaCCL3 ENSG00000006075 chr17
mqvstaalavllctmalcNQFSASLAADTPTACCFSYTSRQIPQNFIADYFETSSQCSKPGVIFLTKRSR
QVCADPSEEWVQKYVSDLELSA

>hsaCCL4 ENSG00000129277 chr17
mklcvtvlsllmlvaafcspalsAPMGSDPPTACCFSYTARKLPRNFVVDYYETSSLCSQPAVVFQTKRS
KQVCADPSESWVQEYVYDLELN

>hsaCCL3L3 ENSG00000256515 chr17
mqvstaalavllctmalcnqvlsAPLAADTPTACCFSYTSRQIPQNFIADYFETSSQCSKPSVIFLTKRG
RQVCADPSEEWVQKYVSDLELSA

>hsaCCL4L1 ENSG00000205020 chr17
mklcvtvlsllvlvaafcslalsAPMGSDPPTACCFSYTARKLPRNFVVDYYETSSLCSQPAVVFQTKRG
KQVCADPSESWVQEYVYDLELN

>hsaCCL3L1 ENSG00000205021 chr17
mqvstaalavllctmalcnqvlsAPLAADTPTACCFSYTSRQIPQNFIADYFETSSQCSKPSVIFLTKRG
RQVCADPSEEWVQKYVSDLELSA

>hsaCCL4L2 ENSG00000197262 chr17
mklcvtvlsllvlvaafcslalsAPMGSDPPTACCFSYTARKLPRNFVVDYYETSSLCSQPAVVFQTKRG
KQVCADPSESWVQEYVYDLELN

>hsaCCL22 ENSG00000102962 chr16
mdrlqtallvvlvllavalqateaGPYGANMEDSVCCRDYVRYRLPLRVVKHFYWTSDSCPRPGVVLLTF
RDKEICADPRVPWVKMILNKLSQ

>hsaCX3CL1 ENSG00000006210 chr16
mapislswllrlatfchltvllagQHHGVTKCNITCSKMTSKIPVALLIHYQQNQASCGKRAIILETRQH
RLFCADPKEQWVKDAMQHLDRQAAALTRNGGTFEKQIGEVKPRTTPAAGGMDESVVLEPEATGESSSLEP
TPSSQEAQRALGTSPELPTGVTGSSGTRLPPTPKAQDGGPVGTELFRVPPVSTAATWQSSAPHQPGPSLW
AEAKTSEAPSTQDPSTQASTASSPAPEENAPSEGQRVWGQGQSPRPENSLEREEMGPVPAHTDAFQDWGP
GSMAHVSVVPVSSEGTPSREPVASGSWTPKAEEPIHATMDPQRLGVLITPVPDAQAATRRQAVGLLAFLG
LLFCLGVAMFTYQSLQGCPRKMAGEMAEGLRYIPRSCGSNSYVLVPV

>hsaCCL17 ENSG00000102970 chr16
maplkmlalvtlllgaslqhihaARGTNVGRECCLEYFKGAIPLRKLKTWYQTSEDCSRDAIVFVTVQGR
AICSDPNNKRVKNAVKYLQSLERS

>hsaCCL27 ENSG00000213927 chr9
mkgpptfcsllllsllllspdptaaFLLPPSTACCTQLYRKPLSDKLLRKVIQVELQEADGDCHLQAFVLH
LAQRSICIHPQNPSLSQWFEHQERKLHGTLPKLNFGMLRKMG

>hsaCCL19 ENSG00000172724 chr9
malllalsllvlwtspaptlsGTNDAEDCCLSVTQKPIPGYIVRNFHYLLIKDGCRVPAVVFTTLRGRQL
CAPPDQPWVERIIQRLQRTSAKASLALPGPVSSL

>hsaCCL21 ENSG00000137077 chr9

```
maqslalsllilvlafgiprtqgSDGGAQDCCLKYSQRKIPAKVVRSYRKQEPSLGCSIPAILFLPRKRS
QAELCADPKELWVQQLMQHLDKTPSPQKPAQGCRKDRGASKTGKKGKGSKGCKR

>hsaCCL26       ENSG00000006606         chr7
mmglslasavllasllslhlgtatrgSDISKTCCFQYSHKPLPWTWVRSYEFTSNSCSQRAVIFTTKRGK
KVCTHPRKKWVQKYISLLKTPKQL

>hsaCCL24       ENSG00000106178         chr7
maglmtivtsllflgvcaHHIIPTGSVVIPSPCCMFFVSKRIPENRVVSYQLSSRSTCLKAGVIFTTKKG
QQFCGDPKQEWVQRYMKNLDAKQKKASPRARAVAVKGPVQRYPGNQTTC

>hsaCCL20       ENSG00000115009         chr2
mcctksllllaalmsvlllhlcgeseaASNFDCCLGYTDRILHPKFIVGFTRQLANEGCDINAIIFHTKKK
LSVCANPKQTWVKYIVRLLSKKVKNM

>hsaCCL25       ENSG00000131142         chr19
mnlwllaclvagflgawapavhtQGVFEDCCLAYHYPIGWAVLRRAWTYRIQEVSGSCNLPAAIFYLPKR
HRKVCGNPKSREVQRAMKLLDARNKVFAKLHHNTQTFQAGPHAVKKLSSGNSKLSSSKFSNPISSSKRNV
SLLISANSGL

>hsaCCL28       ENSG00000151882         chr5
mqqrglaivalavcaalhaseaILPIASSCCTEVSHHISRRLLERVNMCRIQRADGDCDLAAVILHVKRR
RICVSPHNHTVKQWMKVQAAKKNGKGNVCHRKKHHGKRNSNRAHQGKHETYGHKTPY

>hsaXCL2        ENSG00000143185         chr1
mrllilallgicsltayivegVGSEVSHRRTCVSLTTQRLPVSRIKTYTITEGSLRAVIFITKRGLKVCA
DPQATWVRDVVRSMDRKSNTRNNMIQTKPTGTQQSTNTAVTLTG

>hsaXCL1        ENSG00000143184         chr1
mrllilallgicsltayivegVGSEVSDKRTCVSLTTQRLPVSRIKTYTITEGSLRAVIFITKRGLKVCA
DPQATWVRDVVRSMDRKSNTRNNMIQTKPTGTQQSTNTAVTLTG



>mmuCxcl5       ENSMUSG00000029371      chr5
mslqlrssaripsgsispfmrmaplaflllftlpqhlaeaAPSSVIAATELRCVCLTVTPKINPKLIANL
EVIPAGPQCPTVEVIAKLKNQKEVCLDPEAPVIKKIIQKILGSDKKKAKRNALAVERTASVQ

>mmuCxcl7       ENSMUSG00000029372      chr5
mgfrlrptssctracplhnlqilllllglilvalapltagKSDGMDPYIELRCRCTNTISGIPFNSISLVN
VYRPGVHCADVEVIATLKNGQKTCLDPNAPGVKRIVMKILEGY

>mmuCxcl4       ENSMUSG00000029373      chr5
msvaavfrglrpspelllllgllflpavvaVTSAGPEESDGDLSCVCVKTISSGIHLKHITSLEVIKAGRH
CAVPQLIATLKNGRKICLDRQAPLYKKVIKKILES

>mmuCxcl3       ENSMUSG00000029379      chr5
mapptcrllsaalvlllllatnhqatgAVVASELRCQCLNTLPRVDFETIQSLTVTPPGPHCTQTEVIAT
LKDGQEVCLNPQGPRLQIIIKKILKSGKSS

>mmuCxcl15      ENSMUSG00000029375      chr5
maaqgwsmlllavlnlgifvrpcdtQELRCLCIQEHSEFIPLKLIKNIMVIFETIYCNRKEVIAVPKNGS
MICLDPDAPWVKATVGPITNRFLPEDLKQKEFPPAMKLLYSVEHEKPLYLSFGRPENKRIFPFPIRETSR
HFADLAHNSDRNFLRDSSEVSLTGSDA

>mmuCxcl1       ENSMUSG00000029380      chr5
mipatrsllcaallllatsrlatgAPIANELRCQCLQTMAGIHLKNIQSLKVLPSGPHCTQTEVIATLKN
```

GREACLDPEAPLVQKIVQKMLKGVPK

>mmuCxcl2 ENSMUSG00000058427 chr5
mapptcrllsaalvllllllatnhqatgAVVASELRCQCLKTLPRVDFKNIQSLSVTPPGPHCAQTEVIAT
LKGGQKVCLDPEAPLVQKIIQKILNKGKAN

>mmuCxcl9 ENSMUSG00000029417 chr5
mksavlfllgiifleqcgvrgTLVIRNARCSCISTSRGTIHYKSLKDLKQFAPSPNCNKTEIIATLKNGD
QTCLDPDSANVKKLMKEWEKKISQKKKQKRGKKHQKNMKNRKPKTPQSRRRSRKTT

>mmuCxcl10 ENSMUSG00000034855 chr5
mnpsaavifclillglsgtqgIPLARTVRCNCIHIDDGPVRMRAIGKLEIIPASLSCPRVEIIATMKKND
EQRCLNPESKTIKNLMKAFSQKRSKRAP

>mmuCxcl11 ENSMUSG00000060183 chr5
mnrkvtaialaaiiwataaqgFLMFKQGRCLCIGPGMKAVKMAEIEKASVIYPSNGCDKVEVMCLDPRSK
QARLIMQAIEKKNFLRRQNM

>mmuCxcl13 ENSMUSG00000023078 chr5
mrlstatllllllasclspghgILEAHYTNLKCRCSGVISTVVGLNIIDRIQVTPPGNGCPKTEVVIWTKM
KKVICVNPRAKWLQRLLRHVQSKSLSSTPQAPVSKRRAA

>mmuCxcl12 ENSMUSG00000061353 chr6
mdakvvavlalvlaalcisdgKPVSLSYRCPCRFFESHIARANVKHLKILNTPNCALQIVARLKNNNRQV
CIDPKLKWIQEYLEKALNKRLKM

>mmuCxcl14 ENSMUSG00000021508 chr13
mrllaaallllllalcasrvdgSKCKCSRKGPKIRYSDVKKLEMKPKYPHCEEKMVIVTTKSMSRYRGQE
HCLHPKLQSTKRFIKWYNAWNEKRRVYEE

>mmuCxcl16 ENSMUSG00000018920 chr11
mrrgfgplslafflfllalltlpgdgNQGSVAGSCSCDRTISSGTQIPQGTLDHIRKYLKAFHRCPFFIR
FQLQSKSVCGGSQDQWVRELVDCFERKECGTGHGKSFHHQKHLPQASTQTPEAAEGTPSDTSTPAHSQST
QHSTLPSGALSLNKEHTQPWEMTTLPSGYGLEARPEAEANEKQQDDRQQEAPGAGASTPAWVPVLSLLAI
VFFLTAAMAYVLCNRRATQQNSAGLQLWYTPVEPRP

>mmuCxcl17 ENSMUSG00000060188 chr7
mkllaspfllllpvmlmsmvfsSPNPGVARSHGDQHLAPRRWLLEGGQECECKDWFLQAPKRKATAVLGP
PRKQCPCDHVKGREKKNRHQKHHRKSQRPSRACQQFLKRCHLASFALPL

>mmuCcl2 ENSMUSG00000035385 Chr11
mqvpvmllgllftvagwsihvlaQPDAVNAPLTCCYSFTSKMIPMSRLESYKRITSSRCPKEAVVFVTKL
KREVCADPKKEWVQTYIKNLDRNQMRSEPTTLFKTASALRSSAPLNVKLTRKSEANASTTFSTTTSSTSV
GVTSVTVN

>mmuCcl7 ENSMUSG00000035373 Chr11
mrisatllclllliaaafsiqvwaQPDGPNASTCCYVKKQKIPKRNLKSYRRITSSRCPWEAVIFKTKKGM
EVCAEAHQKWVEEAIAYLDMKTPTPKP

>mmuCcl11 ENSMUSG00000020676 Chr11
mqsstallfllltvtsftsqvlaHPGSIPTSCCFIMTSKKIPNTLLKSYKRITNNRCTLKAIVFKTRLGK
EICADPKKKWVQDATKHLDQKLQTPKP

>mmuCcl12 ENSMUSG00000035352 Chr11
mkistllcllliattispqvlaGPDAVSTPVTCCYNVVKQKIHVRKLKSYRRITSSQCPREAVIFRTILD
KEICADPKEKWVKNSINHLDKTSQTFILEPSCLG

>mmuCcl8 ENSMUSG00000009185 Chr11
mkiyavllcllliavpvspEKLTGPDKAPVTCCFHVLKLKIPLRVLKSYERINNIQCPMEAVVFQTKQGM

SLCVDPTQKWVSEYMEILDQKSQILQP

>mmuCcl1 ENSMUSG00000020702 Chr11
mkptamalmclllaavwiqdvdsKSMLTVSNSCCLNTLKKELPLKFIQCYRKMGSSCPDPPAVVFRLNKG
RESCASTNKTWVQNHLKKVNPC

>mmuCcl5 ENSMUSG00000035042 chr11
mkisaaaltiiltaaalctpapaSPYGSDTTPCCFAYLSLALPRAHVKEYFYTSSKCSNLAVVFVTRRNR
QVCANPEKKWVQEYINYLEMS

>mmuCcl9 ENSMUSG00000019122 chr11
mkpfhtalsflilttalgiwaqithaTETKEVQSSLKAQQGLEIEMFHMGFQDSSDCCLSYNSRIQCSRF
IGYFPTSGGCTRPGIIFISKRGFQVCANPSDRRVQRCIERLEQNSQPRTYKQ

>mmuCcl6 ENSMUSG00000018927 chr11
mrnsktaisffilvavlgsqaGLIQEMEKEDRRYNPPIIHQGFQDTSSDCCFSYATQIPCKRFIYYFPTS
GGCIKPGIIFISRRGTQVCADPSDRRVQRCLSTLKQGPRSGNKVIA

>mmuCcl3 ENSMUSG00000000982 chr11
mkvsttalavllctmtlcnqvfsAPYGADTPTACCFSYSRKIPRQFIVDYFETSSLCSQPGVIFLTKRNR
QICADSKETWVQEYITDLELNA

>mmuCcl4 ENSMUSG00000018930 chr11
mklcvsalslllvaafcapgfsAPMGSDPPTSCCFSYTSRQLHRSFVMDYYETSSLCSKPAVVFLTKRG
RQICANPSEPWVTEYMSDLELN

>mmuCcl22 ENSMUSG00000031779 chr8
matlrvpllvalvllavaiqtsdaGPYGANVEDSICCQDYIRHPLPSRLVKEFFWTSKSCRKPGVVLITV
KNRDICADPRQVWVKKLLHKLS

>mmuCx3cl1 ENSMUSG00000031778 chr8
mapsplawllrlaaffhlctllPGQHLGMTKCEIMCDKMTSRIPVALLIRYQLNQESCGKRAIVLETTQH
RRFCADPKEKWVQDAMKHLDHQAAALTKNGGKFEKRVDNVTPGITLATRGLSPSALTKPESATLEDLALE
LTTISQEARGTMGTSQEPPAAVTGSSLSTSEAQDAGLTAKPQSIGSFEAADISTTVWPSPAVYQSGSSSW
AEEKATESPSTTAPSPQVSTTSPSTPEENVGSEGQPPWVQGQDLSPEKSLGSEEINPVHTDNFQERGPGN
TVHPSVAPISSEETPSPELVASGSQAPKIEEPIHATADPQKLSVLITPVPDTQAATRRQAVGLLAFLGLL
FCLGVAMFAYQSLQGCPRKMAGEMVEGLRYVPRSCGSNSYVLVPV

>mmuCcl17 ENSMUSG00000031780 chr8
mktftsafgtmrslqmlllaalllgtflqharaARATNVGRECCLDYFKGAIPIRKLVSWYKTSVECSRD
AIVFLTVQGKLICADPKDKHVKKAIRLVKNPRP

>mmuCcl27a ENSMUSG00000073888 chr4
meglspasslplllllllspapeaaLPLPSSTSCCTQLYRQPLPSRLLRRIVHMELQEADGDCHLQAVVLH
LARRSVCVHPQNRSLARWLERQGKRLQGTVPSLNLVLQKKMYSNPQQQN

>mmuCcl21b ENSMUSG00000094065 chr4
maqmmtlsllslvlalcipwtqgSDGGGQDCCLKYSQKKIPYSIVRGYRKQEPSLGCPIPAILFLPRKHS
KPELCANPEEGWVQNLMRRLDQPPAPGKQSPGCRKNRGTSKSGKKGKGSKGCKRTEQTQPSRG

>mmuCcl19b ENSMUSG00000095348 chr4
mlprlasiswahvilhlsfppaptlgGANDAEDCCLSVTQRPIPGNIVKAFRYLLNEDGCRVPAVVFTTL
RGYQLCAPPDQLWVDRIIRRLKTSSAKASLTLLSPASTLPTPRDSSS

>mmuCcl21d ENSMUSG00000073878 chr4
maqmmtlsllslvlalcipwtqgSDGGGQDCCLKYSQKKIPYSIVRGYRKQEPSLGCPIPAILFLPRKHS
KPELCANPEEGWVQNLMRRLDQPPAPGKQSPGCRKNRGTSKSGKKGKGSKGCKRTEQTQPSRG

>mmuCcl27c ENSMUSG00000073877 chr4

meglspasslplllllllspapeaaLPLPSSTSCCTQLYRQPLPSRLLRRIVHMELQEADGDCHLQAVVLH
LARRSVCVHPQNRSLARWLERQGKRLQGTVPSLNLVLQKKMYSHPQQQN

>mmuCcl21c ENSMUSG00000095675 chr4
maqmmtlsllslvlalcipwtqgSDGGGQDCCLKYSQKKIPYSIVRGYRKQEPSLGCPIPAILFLPRKHS
KPELCANPEEGWVQNLMRRLDQPPAPGKQSPGCRKNRGTSKSGKKGKGSKGCKRTEQTQPSRG

>mmuCcl19c blastsearch chr4
mlprlasiswahvilhlsfppaptlgGANDAEDCCLSVTQRPIPGNIVKAFRYLLNEDGCRVPAVVFTTL
RGYQLCAPPDQPWVDRIIRRLKKSSAKASLTLLSPASTLPTPRDSSS

>mmuCcl27b ENSMUSG00000096826 chr4
mmeglspasslplllllllspapeaaLPLPSSTSCCTQLYRQPLPSRLLRRIVHMELQEADGDCHLQAVVL
HLARRSVCVHPQNRSLARWLERQGKRLQGTVPSLNLVLQKKMYSHPQQQN

>mmuCcl21e ENSMUSG00000096596 chr4
maqmmtlsllslvlalcipwtqgSDGGGQDCCLKYSQKKIPYSIVRGYRKQEPSLGCPIPAILFLPRKHS
KPELCANPEEGWVQNLMRRLDQPPAPGKQSPGCRKNRGTSKSGKKGKGSKGCKRTEQTQPSRG

>mmuCcl19 ENSMUSG00000071005 chr4
maprvtpllafsllvlwtfpaptlgGANDAEDCCLSVTQRPIPGNIVKAFRYLLNEDGCRVPAVVFTTLR
GYQLCAPPDQPWVDRIIRRLKKSSAKNKGNSTRRSPVS

>mmuCcl21a ENSMUSG00000094686 chr4
maqmmtlsllslvlalcipwtqgSDGGGQDCCLKYSQKKIPYSIVRGYRKQEPSLGCPIPAILFSPRKHS
KPELCANPEEGWVQNLMRRLDQPPAPGKQSPGCRKNRGTSKSGKKGKGSKGCKRTEQTQPSRG

>mmuCcl26 ENSMUSG00000070464 chr5
mffdlgllvllaiflsvqlgvaTCGSSIAMSCCPNFSYYVIPWSWVYSYKFTDKSCTSDGVIFFTKTGKQ
FCVQPGAKWVQRFISLVNTRNHL

>mmuCcl24 ENSMUSG00000004814 chr5
magsativaglllllvacaccifpidsVTIPSSCCTSFISKKIPENRVVSYQLANGSICPKAGVIFITKKG
HKICTDPKLLWVQRHIQKLDAKKNQPSKGAKAVRTKFAVQRRRGNSTEV

>mmuCcl20 ENSMUSG00000026166 chr1
macggkrllflalawvllahlcsqaeaASNYDCCLSYIQTPLPSRAIVGFTRQMADEACDINAIIFHTKK
RKSVCADPKQNWVKRAVNLLSLRVKKM

>mmuCcl25 ENSMUSG00000023235 chr8
mklwlfaclvacfvgawmpvvhaQGAFEDCCLGYQHRIKWNVLRHARNYHQQEVSGSCNLRAVRFYFRQK
VVCGNPEDMNVKRAMRILTARKRLVHWKSASDSQTERKKSNHMKSKVENPNSTSVRSATLGHPRMVMMPR
KTNN

>mmuCcl28 ENSMUSG00000074715 chr13
mqqagltlmavavcvafqtseaILPMASSCCTEVSHHVSGRLLERVSSCSIQRADGDCDLAAVILHVKRR
RICISPHNRTLKQWMRASEVKKNGRENVCSGKKQPSRKDRKGHTTRKHRTRGTHRHEASR

>mmuXcl1 ENSMUSG00000026573 chr1
mrllllltflgvccltpwvvegVGTEVLEESSCVNLQTQRLPVQKIKTYIIWEGAMRAVIFVTKRGLKICA
DPEAKWVKAAIKTVDGRASTRKNMAETVPTGAQRSTSTAITLTG

>rnoCxcl6 ENSRNOG00000002843 chr14
msfqlrssaripsrscssftllaflllftlpqhraqaAPFSAMVATELRCVCLTLAPRINPKMIANLEVI
PAGPHCPKVEVIAKLKNQKDNVCLDPEAPLIKKVIQKILGSENKKTKRNALALVRSASTQ

>rnoCxcl7 ENSRNOG00000002829 chr14
mgfrlrptssctrasslrnlqvllslllvalvplavgKPDNNEPFIELRCRCTNTLSGIPLNSISRVNVF
RPGAHCDNVEVIATLKNGKEVCLDPTAPMIKKIVKKILEGY

>rnoCxcl4 ENSRNOG00000028015 chr14
msaaavfrglrpspellllgllllpavvavtraSPEESDGDLSCVCVKTSSSRIHLKRITSLEVIKAGPH
CAVPQLIATLKNGSKICLDRQVPLYKKIIKKLLES

>rnoCxcl3 ENSRNOG00000028043 chr14
mapptrrllnaalllllllmatsHQPSGTVVARELRCQCLKTLPRVDFENIQSLTVTPPGPHCTQTEVIA
TLKDGQEVCLNPQAPRLQKIIQKLLKSPSL

>rnoCxcl15 EDL88572.1 chr14
maarlwpmfllaifnlgifappcdtEELRCQCIQITSESIPCKFIKTIEVIYENIYCNRMEVIAVLRDGN
AICLHPNSTCVRTLIDNIPSRPFIPKDYASANNLRGMKFMYSVEFEKLLYLSFMEPQV

>rnoCxcl1 ENSRNOG00000002802 chr14
mvsatrsllcaallllatsrqatgAPVANELRCQCLQTVAGIHFKNIQSLKVMPPGPHCTQTEVIATLKN
GREACLDPEAPMVQKIVQKMLKGVPK

>rnoCxcl2 ENSRNOG00000002792 chr14
mapptrqllnavlvlllllatnhqgtgVVVASELRCQCLTTLPRVDFKNIQSLTVTPPGPHCAQTEVIAT
LKDGHEVCLNPEAPLVQRIVQKILNKGKAN

>rnoCxcl9 ENSRNOG00000022242 chr14
mksvalflmgiifldhcgvrgTLVIRNQRCSCISTSQGTFHYKSLKDLKQFAPSPNCNKTEIIATLKNGD
QTCLDPDSARVKKLMKEWEKKISQKKKQKRGKNHQRSKKTRKAKTPHHPESKKTA

>rnoCxcl10 ENSRNOG00000022256 chr14
mnpsaavvlclvllslsgtqgIPLARTVRCTCIDFHEQPLRPRAIGKLEIIPASLSCPHVEIIATMKKNN
EKRCLNPESEAIKSLLKAVSQRRSKRAP

>rnoCxcl11 ENSRNOG00000022298 chr14
mnrtgmavalamiiwattvpgFVMFKGGRCLCIDRGVKVVKMAAIKEVSVIYPSNGCDKVEVIVTLKAHK
GQRCLDPTSKQARLIMQTIQKKNFLRRQNM

>rnoCxcl13 ENSRNOG00000024899 chr14
mrlctaalllllaiclppghgILETHYTNLKCRCSKVSSTFINLILVDWIQVIRPGNGCPKTEIIFWTKA
KKAICVNPTARWLPKVLKFVRRSITSTPQAPVSKKRAA

>rnoCxcl12 ENSRNOG00000013589 chr4
mdakvvavlalvlaalcisdgKPVSLSYRCPCRFFESHVARANVKHLKILNTPNCALQIVARLKSNNRQV
CIDPKLKWIQEYLDKALNKRLKM

>rnoCxcl14 ENSRNOG00000011984 NP_001013155.1 chr17
mrllaaallllllalcasrvdgSKCKCSRKGPKIRYSDVKKLEMKPKYPHCEEKMVIITTKSMSRYRGQE
HCLHPKLQSTKRFIKWYNAWNEKRRVYEE

>rnoCxcl16 ENSRNOG00000026647 chr10
mrrgfgplaltlflfffalltlpgdgNQGSVAGSCYCDRTIPSGTQIPPATLNHMRKYLKTFHRCQFFIR
FQLQSKSVCGGSQDQWVRELVNCFEHKQCGIGHGQSFHHQKHVPQASTRIPEATEGKPPDTSTAVQFQST
QQSTFPSGAPSLNKELTRHWETTILPSGYGLEARPVAEANEKQHKQQKEPGAGAGTQALVPVLSLLAIVF
FLVAAMVCVLCNRRVTRQSSSGLQLCYTPVEPRPQGL

>rnoCxcl17 ENSRNOG00000037814 chr1
mkllaspfllllltgmftatvssSPNQEVARHHGDQHQAPRRWLWEGGQECDCKDWSLRVSKRKTTAVLEP
PRKQCPCDHVKGSEKKNRRQKHHRKSQRPSRTCQQFLKRCQLASFTLPL

```
>rnoCcl2         ENSRNOG00000007159      Chr10
mqvsvtllgllftvaaCSIHVLSQPDAVNAPLTCCYSFTGKMIPMSRLENYKRITSSRCPKEAVVFVTKL
KREICADPNKEWVQKYIRKLDQNQVRSETTVFYKIASTLRTSAPLNVNLTHKSEANASTLFSTTTSSTSV
EVTSMTEN

>rnoCcl7         ENSRNOG00000000239      Chr10
mqisaallcvlltaaaftvhvwaQPDGTNSSTCCYVKKQKIPKRNLKSYRKITSSRCPWEAVIFKTKKGM
EVCAEAHQKWVEEAIAYLDMKTSTPKP

>rnoCcl11        ENSRNOG00000007335      Chr10
mqlstallflllltatsftsqvlaHPGSIPTSCCFTMTSKKIPNTLLKSYKRITNNRCTLKAIVFKTKLGK
EICADPKKKWVQDATKHLDQKLQTPKP

>rnoCcl12        ENSRNOG00000029768      Chr10
mkistllcllliaaaispqvlaGPDSVFTPVTCCYNVAKQKIHIRRLKSYRKITSSQCPREAVIFRTVLD
KELCADPKEKWVKDSMNHLDQKSRTQHP

>rnoCcl1         ENSRNOG00000021851      Chr10
mkllnmvlvcllvaamwlqnvdsKSMHVVSSRCCLNTLENKIALKFIKCYKEIGPSCPYYPAVIFRLIKG
RESCALTNTTWVQDYLKKVKPC

>rnoCcl5         ENSRNOG00000010906      chr10
mkistaasltvilvaaalctpapaSPYGSDTTPCCFAYLSLALPRAHVKEYFYTSSKCSNLAVVFVTRRN
RQVCANPEKKWVQEYINYLEMS

>rnoCcl9         ENSRNOG00000028548      chr10
mkpfhtalsflifaatlgiqaqivhaTETREVLKAEQGHQLFSLGFQDSSDCCLSYTSRVHCSRYKNYFP
TSGGCTKPAIIFVSKKGTLVCANPSDLRVQKCIKRLEQNSQPQIYKQ

>rnoCcl6         ENSRNOG00000030021      chr10
mrhsktaisffilvavlgsqaGLIQDTVKEDRPFNPTIIHQGFQDSSDCCFSYASQIPCSRFIYYFPTSG
GCTKPGIIFVTRKRKRVCANPSDQRVQTCISTLKLGPRSGNSAIA

>rnoCcl3         ENSRNOG00000011205      chr10
mkvstaalavllctmalwnevfsAPYGADTPTACCFSYGRQIPRKFIADYFETSSLCSQPGVIFLTKRNR
QICADPKETWVQEYITELELNA

>rnoCcl4         ENSRNOG00000011406      chr10
mklyvsafsllllvaafcdsvlsAPIGSDPPTSCCFSYTSRKIHRNFVMDYYETSSLCSQPAVVFLTKKG
RQICADPSEPWVNEYVNDLELN

>rnoCcl22        ENSRNOG00000016535      chr19
maslrvpllvalvllavalqtsdaGPYGANVEDSICCQDYIRHPLPPRFVKEFYWTSKSCRKPGVVLITI
KNRDICADPRMLWVKKILHKLA

>rnoCx3cl1       ENSRNOG00000016326      chr19
mapsqlawllrlaaffhlctllagQHLGMTKCNITCHKMTSPIPVTLLIHYQLNQESCGKRAIILETRQH
RHFCADPKEKWVQDAMKHLDHQTAALTRNGGKFEKRVDNVTPRITSTTRGLSPTALAKPESATVEDLTLE
PTAISQEARRPMGTSQEPPAAVTGSFPSTSKAQDAGLAAKPQSTGISEVAAVSTTIWPSSAVYQSGSSLW
AEEKATESPPTIALSTQVSTTSSPKQNVGSEGQPPWVQEQDSTPEKSPGPEETNPVHTDIFQDRGPGSTV
HPSVAPTSSEKTPSPELVASGSQAPKVEEPIHATADPQKLSVFITPVPDSQAATRRQAVGLLAFLGLLFC
LGVAMFAYQSLQGCPRKMAGEMVEGLRYVPRSCGSNSYVLVPV

>rnoCcl17        ENSRNOG00000016278      chr19
mmslqmlllaalllgtslqhasaARATNVGRECCLDYFKGAIPIRKLVTWFRTSVECPRDAIVFETVQGR
LICTDPKDKHVKKAIRHLKNQRL

>rnoCcl27        ENSRNOG00000039530      chr5
meglsltgslplillllspvpeaALPLPSSTSCCTQLYRQPLPTRLLRRIVRVELQEADGDCHLQAVVLH
```

LTRRSVCVHPQNRSLARWLERHGKRLQGTLPSLNPVLQKKMCPSPQKQK

rnoCcl19 ENSRNOG00000015668 chr5
masrvtpllafsllvlwtfsapalgGANDAEDCCLSVTQRPIPGNIVKAFRYLLIQDGCRVPAVVFTTLR
GYQLCAPLDQPWVERIIRRLKKSSSKSKGGSTKRGPVS

>rnoCcl21 ENSRNOG00000034290 chr5
maqmlalsllslvlvlcvpwtqgSDGGGQDCCLRYSQKKIPYNIVRGYRKQEPSLGCPIPAILFAPRKQS
QPELCANPEEAWVQKLMRRLDQPPAPGKQNCRKNRGTSKSGKKGRGSKGCKGTEQTDSKRVSQ

>rnoCcl26 ENSRNOG00000001444 chr12
mktfslgpivllavflsvqlgvatcESEMPMPCCSYFSHYVIPWHWLYSYQITDSSCPNEGVIFTTKTGK
QYCVQPGDKWVQRYISLLNAKKHW

>rnoCcl24 ENSRNOG00000031162 chr12
magsttiiaslllllvactccICPTGSVTIPSSCCVTFISKKIPVNRVISYQLANGSICPKAGVIFITKKG
HKICTDPKLPWVQKHIKNLDAKRNQPSEGAKALGPKFVIQKLRGNSTKV

>rnoCcl20 ENSRNOG00000015992 chr9
mackhlpflalagvllaylcsqseaASNFDCCLTYTKNVYHHARNFVGFTTQMADEACDINAIIFHLKSK
RSVCADPKQIWVKRILHLLSLRTKKM

>rnoCcl25 ENSRNOG00000028531 chr12
mklqlfaclaacfvgawvpvvraQGAFEDCCLGYQPRIKWNVLRHARHYHQQEVSGSCNLRAVIFYFRQK
VVVCGNPEDKDVKRAVKILTARKQPVHSPQKSTSDYPTERKKSNYMKFKVENPKGTRMRNATLGHPRMVM
MSRKTNN

>rnoCcl28 ENSRNOG00000017547 chr2
mqqagltlvavavcvafrtseaILPIASSCCTEVSHHIPRRLLERVNSCSIQRADGDCDLAAVILHVKRR
RICVSPHNPTLKRWMSASEMKNGKENLCPRKKQDSGKDRKGHTPRKHGKHGTRRIHGTHGHEAPR

>rnoXcl1 ENSRNOG00000002964 chr13
mrllllltflgvccfaawvvegVGTEVLQESICVSLRTQRLPVQKIKTYTIKEGAMRAVIFVTKRGLRICA
DPQAKWVKTAIKTVDGRSSASKSKAETIPTQAQRSASTAIALSG

>ocuCxcl8 ENSOCUG00000011835 chr15
mnsklavallatfllsltlceaAVLTRIGTELRCQCIKTHSTPFHPKFIKELRVIESGPHCANSEIIVKL
VDGRELCLDPKEKWVQKVVQIFLKRFKNKTQQQ

>ocuCxcl6 ENSOCUG00000011838 chr15
msllssrasrlsgpssclcallalllltppaplasaGPVAAVVRELRCVCLSTTAIHPKMIARLQVIAAG
PQCSKVEVVASLKNGKEICLDPEAPLIKKAIQKILESGNKEN

>ocuCxcl4 ENSOCUG00000017941 chr15
msipeasgaprprpsrgllllglllllltvaaaASDDPKESEGDLHCVCVKTTSLVRPRHITNLELIKAGA
HCPTAQLITLKNGRKLCLDQAALYKKVIKKLLKE

>ocuCxcl3 ENSOCUG00000024425 chr15
PALTELRCQCLQTVQGIHLKNIQNLKVLSPGPHCAQTEVIATLKSGQEACLNPAAPMVKKFLQKRLS

>ocuCxcl15 Blast search chr15
malgsspaplllaalvlgifadfyesQELRCQCIQIYSNFISPKLIKNVQMIPSGPYCSTKEVIVTLKDG
RLICLDPEAEWVMTLIKNTGD

>ocuCxcl1 NP_001075855.1 chr15

mapaacpaaapsgprflrtamllllllaasrraagAAALTELRCQCLQTVQGIHLKSIQSLKVLSPGPHC
AQTEVIATLKSGQEACLNPAAPMVKKFLQKRLSKGSSN

>ocuCxcl2   ENSOCUG00000024883   chr15
mapaacpaasrfprllrtallllllllaasrraagAPALTELRCQCLQTVQGIHLKSIQSLKVLSPGPHCA
QTEVIATLKSGQEACLNPASPMVKKLLQKMLSKSNTS

>ocuCXCL9   ENSOCUG00000014671   chr15
mkkscvlfllgftflvligvqgSPIMRNGRCSCISSTQGKIHLQSLKDLKQFSPSPSCGKTEIIATKKDG
TQICLNPDSTEVKELVEKWKKQSSPKKKQKKGKKQRKVKKSLKKSQRPHQKKTA

>ocuCXCL10   ENSOCUG00000016280   chr15
mnqsailifcliflltlsgtkgMPLSRTVRCTCINISNKPVNPRSLEKLEIIPASQSCANVEIIATMKKDG
EKRCLNPELKAIKKLLKAFSKERSRGSS

>ocuCXCL11   ENSOCUG00000026515   chr15
msvkgvllvlavilcattvqgFPMFKGGRCLCTGPGVKAVKVADVQKATIIYPSGSCDKTEVIITLKAKK
GQQCLNPRSKQASLIIKQIERKTLRNQNV

>ocuCXCL13   ENSOCUG00000024738   chr15
mrltsasllftllvsslspaqgVLEAYNTDLKCECVQETANYIPIRFIDRLQIVPPGNGCPKREVIVWLK
TKIAVCVKPHAKWLQKLIQILQKKTMLSTPPAPVI

>ocuCXCL16   ENSOCUG00000027827   chr19
msrrqvrllllllaalalsvsaNEGSVTGSCHCDQIISSSSPPNPKLMEHFRKHLRAYHRCTAYIRFRLHS
RSVCGGNHDPWVHELLRCFDRGECGRAHWESLAHRKPLPSRSTQIPKYTEEASAHLPSTAQTHLPPTLPP
GAHANKTTTAPPSQSLEVGPEAGQNQKQLEEIVGPAAGTSAMVPVLTLLAITFLLTGVLMYVLCKRRRGR
SPKYSSGKP..

>ocuCXCL17   ENSOCUG00000021666   Scaffold GL018789
mapekvlipsllllllslllmptvssTPHSGVARGHGDQRQTSGRWLWEDGQECECKGWFLRAPKRKLMTV
PRLPKKHCPCDHFKGTVKKTRHQKSHRQPNKHSRACQQFLKRCQLASFALPL

>ocuCcl2   ENSOCUG00000021710   Chr19
mkvsatllcllliavafsshvlaQPDAVNSPVTCCYTFTSKTISVKRLMSYRRINSTKCPKEAVIFMTKL
AKGICADPKQKWVQDAIANLDKKMQTPKTLTSYSTTQEHTTNLSSTRTPSTTTSL

>ocuCcl7   Blast search   Chr19
mqisaallcllltvaafssqvlaQPEGTNSGKTCCYRFHNRRMDPQKLRSYTLISISYCPREAVIFKTKQ
HREVCADPKWPWAQNAIAYLNKKTQTSKP

>ocuCcl11   ENSOCUG00000005935   Chr19
mkvsaallcmlvtaavfssqvlaQPAFVPTTCCFSMAKKMPLQRLESYRRISGSKCPQKAVIFKTKLAKE
ICADPKEKWVQDSIKYLDQKSKTSKP

>ocuCcl13   ENSOCUG00000013412   Chr19
mkvsaallcllllaaacssqalaQTETKPALTACCFSFVRKRIPLQRLVSYRKTSKACVKEAVIFRTRRD
QELCADPMQKWVQDTMRVL

>ocuCcl1   ENSOCUG00000013416   Chr19
mklvpvalaclllvamwpqdvdgKSMHVSSSNCCFSFVEKRIPLKTIHCYRRSSSTCPYDAIIFKLRGGR
ESCALKTVGWLQGYFKKMKPCLPKGP

>ocuCCL5   ENSOCUG00000002848   chr19
mevsaaalavlltaaalcapasaSPYASDTTPCCFAYISRLLPRAHVTEYFYTSGKCSFPAVVFVTRKNR
QVCANPEKKWVREYINSLEMS

>ocuCCL14   ENSOCUG00000002849   chr19
mkvsvaaipllllfitpialgSRAEFSPRGPYHPAECCFSYITRVVPRQRITDYYETSSECSKPGIVFIT

KKGYSICANPRDDWVQDYIRDLEK

>ocuCCL15 blastsearch chr19
mklstaalslllla avlgtqaFVLHNTETRQLTRAMDRLINRRPIQGVNPIQSIMPLSSNVRPSDCCYGY
TTRPLQCSVMEDFFETNSQCSMPAVIFITKCGKLICANPRNNEVEECMETLKTQGTRLGPRAAAGLLY

>ocuCCL23 ENSOCUG00000000686 chr19
mkvsvaalsflalilaaalgsqaLVTHDTDTKQLALVVDRFDKEIVQGFHRPTDCCFSYTKRNIQCFLMQ
DYIETSSQCSMPAVIFLTKRGQKVCADSSDERVRKCVSSLQRDLPTRNL

>ocuCCL3 ENSOCUG00000000771 chr19
mkvsgvalavllcamalstqvfsIPLGADTPTACCFSYISRQIPYKFIADYFETSSQCSKPGVIFLTKRG
RQVCADISEAWVQGYINDLELNS

>ocuCCL4 ENSOCUG00000026206 chr19
mklgvtvlsvallvaalcppahsAPMGSDPPTACCFSYTLRQLPRHFVIDYFETSSLCSQPAVVFQTKKG
RQVCANPSESWVQEYVDDLELN

>ocuCCL22 ENSOCUG00000015478 chr5
masmqapllaalmllavvlqateaGPYGANVEDSVCCRDYMRRPLAPRVVKYFYWTSDSCRRPGVVLVTL
KGLEICADPRLPWVKKILRKLEQ

>ocuCX3CL1 ENSOCUG00000007482 chr5
mapiapqwllrlaafcplmvplagQHLGVAKCSIICNNMTPKIPVALLISYQWNQESCGKPAIVLETKRH
KRFCADPQAPWVQEAMKHLDRQAVARTPNSGTFEKQIGAVKPGASTVKPGTTAAPGGMPEPAVSEPRPTG
DSRSPEPTPAPQEAQTTLGTSPALPAGVPSSSGSGSLVTQKPQDGRPPVGPTGPEFVNTAPVSTAGSWQT
SAHQHGATESPSAETPSTEHPSTQALTTSYPASEENLGSESQSPGAPALSPRPGDPQSTWETVPVVAHTG
AFQDGGAGSTAYASVVPGSSEGTPSREPAASGSWTPKAEEPVHATVDPQRLGVLITPVPDSQAATRRQAV
GLLAFLGLLFCLGVAMFAYQSLQGCPRKMAGEMVEGLRYVPRSCGANSYVLVPV

>ocuCCL27 ENSOCUG00000001224 chr1
megcsptsrllllllllsldpgavliLPPSTTCCTQLYRQPLSNKLLRKVIRVELQEADGDCHLQAFVLH
LARRSVCIHPQNRSLARWFERQGKRLQGTQPDFWVARENGLEPHKAPIKQH

>ocuCCL19 ENSOCUG00000001515 chr1
mapgaaqllalsllvlwtslapalgGANDAEDCCLSVTQRPIPSNIVRAFRYLLVTDGCRVPAVVFTTLR
GFQLCAPPEQPWVGRIIRRLKRISAKGKYQSS

>ocuCCL21 ENSOCUG00000016448 chr1
maqplalsllflvlalcipqtqgSDGGAQDCCLKYSLRKIPHKVVRSYRKQEPSLGCPISAILFSPRKRS
QPELCADPKEPWVQQLMKRLDRPPAPKKQVQACRKDGGASKSGKKGKGCKSLPRTEQPQTPKGP

>ocuCCL24 ENSOCUG00000025061 chr6
magpatiaaslllla lsahhftpagaVVIPSSCCMNFIPKKIPESQVVSYQLSSGSVCPRAGVIFTTKKG
YRVCGDPKQQWVQSYVRNLVAKRRKPSTGARAPGGRRPGEMRA

>ocuCCL20 ENSOCUG00000000868 chr7
mtcgsksllls almsllllhlcskleaASNFDCCLRYTECRIHPRFIVGFTQQLANEACDINAIILHTKK
NLSVCADPKKEWVKRVVHIL..

>ocuCCL25 ENSOCUG00000025780 Scaffold GL018767
mnswilaclvvcfvgawtpavhtQVAGISEDCCLAYHSLSWAVLQRTQGYQLQEVSGSCNLRAVRFCLKH
RIVCGNPQDKKVQRAVKLLNTRRKSCHHSQKTLRGVPRSERKKLSSRKSWPPSSKQSSPAGSGKRNATLQ
TAAKLGEDARSRD

>ocuCCL28 ENSOCUG00000007934 chr11
mqcaglllvalatcvalqpseaILPFFSSCCTEVSPHLSRRLLKMVHTCHLQRADGDCDLDAVALYFGRR
RFCVTTENHMLKEWMKAQAAKKIGQRIICREKKYNKSSSRGTSVEGSMTGCHKTSS

```
>ocuXCL1        ENSOCUG00000012952      chr13
mrrlllvllcicclttsivegVGSEVPDKSICVSLTTQPLPVNRIRTYTIKEGPMKAVIFITKRGLKVCA
DPQAKWVKTAIKSISNKPSTRRSTTQTQPTGTQQSTRRAVTLTA


>cpoCxcl8        ENSCPOG00000011103      scaffold_1
mpsqlrvailaafllsavlcEGMVVTKLVSELRCQCIKIHTTPFHPKFIKELKVIESGPRCANSEIIVKL
SDNRQLCLDPKKKWVQDVVSMFLKRTESQDS

>cpoCxcl6        ENSCPOG00000020184      scaffold_1
mypvpgrasrtprllcallalllllpppesrvhaGPVAAARELRCVCVTITPGVHPKMIASLQVTVSGPQC
PNVEVVATLKNGKQICLDPEAPLIKRVIQKMLDSGNKQN

>cpoCxcl7        ENSCPOG00000022396      scaffold_1
cpsarlhrvlgglmllslllaalvpstngQRKRNLGENKATKIIEELAELRCLCLKTISAMHPSKISSVE
VLKAGAHCPKIQVIAKLKDGKKVCLDPDLPGVKKMIQKILAGQGS

>cpoCxcl1        ENSCPOG00000015206      scaffold_1
magaapktlrfapllllllllvlgtsrraagAPAASELRCRCLRPVRGLHPKNIQSVAVTAPGPHCHQTE
VLATLKDGREACLDPEAPMVQKVLQRMLKGSKAT

>cpoCxcl9        XP_003477696.1          scaffold_5
mkghsvsfflgiiflvlfevqgTPVMRKGRCYCINSSDDVISLKSLKDLKQFVPSPSCEKTEIIATKRNG
DQTCLNPDSTKVKKLIKQWEKKVTQKKKQKKAKKYQKNKKIIKVKRSKAPCQEKNT

>cpoCxcl10       ENSCPOG00000002046      scaffold_5
mnhntilifcliflt lsgtqgIPHSRTIRCTCIETSTQPVNPKSFKKLEIIPASQSCPRVEIIATMKMNG
EKRCLDPESKVIKNLLKAVRKERSKRS

>cpoCxcl11       ENSCPOG00000012442      scaffold_5
mnmkglvivlamilsatvvqgFPMFKRGRCLCIGPGLRAVKVADIAKASIIYPSNSCDKIEVIITLKAHK
GQRCLNPRSKQAGLIIKQVERKNSLKH

>cpoCxcl13       ENSCPOG00000001379      XP_003477708.1  scaffold_5
mrlsavalllsvlisslspgqgILEAYNTNLKCRCMQETSQVVRIPNIHRLRILPPGNGCPKTQIIIQLR
NKTLVCLNPAAKWVQQFVARVKSRNFHSTAAAPVMKKVP

>cpoCxcl12       ENSCPOG00000014912      scaffold_62
mdakvvavlalvlaalclgdgKPVSLSYRCPCRFFESHVARANVKHLKILNTPNCALQIVARLKNNNRQV
CIDPKLKWIQEYLEKALNKGRREDKMGKKEKIGKKKRQKKRKAAQKRKN

>cpoCxcl17       ENSCPOG00000021240      scaffold_80
mkvkisfllllplmltsmvssSPKSGVARAHGEQRQASRRWLQEGSRECECKDWFQRALRRKPMTVPAL
PKKQCPCDHLKVNMKKSRHHKHQRKPNKHSRACQEFLKQCQLASIALPL

>cpoCcl2L        XP_003469662.1         scaffold_32:12,677,940-12,679,720
mqgssvflcllvlaatfsslllaQPVGVNTPICCYVFNRKIPLKRVRGYERITSSRCPQEAVIFRTLKNK
EVCADPTQKWVQDYIAKLDQRTQQKQNSTAPPTSKPLNIRFTTQDPKNRS

>cpoCcl2         ENSCPOG00000013468   scaffold_32:12,677,940-12,679,720
mqrssvllcllvieatfcsllmaQPDGVNTPTCCYTFNKQIPLKRVKGYERITSSRCPQEAVIFRTLKNK
EVCADPTQKWVQDYIAKLDQRTQQKQNSTAPQTSKPLNIRFTTQDPKNRS

>cpoCcl7         ENSCPOG00000013035 scaffold_32:12,677,940-12,679,720
mqviavllcllltaanfsslllaQPDGVNISTCCYKRSQRIRVQRLESYTRITSSKCPWQAVIFKTKFNR
EICADPKQQWVQDSMKYIDKKSKTPKS
```

```
>cpoCcl11       ENSCPOG00000013470   scaffold_32:12,677,940-12,679,720
mkvstaflclllltvsafsaqvlaHPGIPSACCFRVTNKKISFQRLKSYKIITSSKCPQTAIVFEIKPDKM
ICADPKKKWVQDAKKYLDQISQTTKP

>cpoCcl13       Blast search         scaffold_32:12,677,940-12,679,720
mqvsasllclmltaaavsvqvlaQADTNETPLACCLTLTHRKIPLNYLKGYELTSDKCPLTAVIFKTRRN
IQICADPTKKWVQHAMVYLNDLKT

>cpoCcl1        ENSCPOG00000002574   scaffold_32: 12,677,940-12,679,720
mkvaavalvclllatvwaKDVDSMSLHVASSRCCYSFVERKISHHRIQSYKGTSSSCPYEAVIFKLKGGR
EICALKTIEWVQDYLTKKKILRLKGK

>cpoCcl5        ENSCPOG00000008852      scaffold_32
mkvsaaalcvilttaalcvpasaSPYASDTTPCCFAYISRALPRTHIKEYFYTSSKCSNLAVVFVTRKNR
QVCANPEKKWVREYINSLEMS

>cpoCcl14       ENSCPOG00000026493      scaffold_32
mkvsmvtislllflitvtleTKTHSSSRGPFYPSECCFSYITHAVLPYRIMGYYETNGDCPKPAVVFITT
KGHRICANPENEWVQDYLKDLEEN

>cpoCcl15       ENSCPOG00000026069      scaffold_32
mkvstaalsflilaavlgsqaQLIYGDKSICNISNMLSFHQPADCCFSYTSRKIQCRLMEDYFRTSSGCP
RPGVIFLTKKGKLVCADPSDRRVQDCIRNLTPTSLPEILDTLKLA

>cpoCcl23       ENSCPOG00000021606      scaffold_32
micmaalsfliiaaalgsqaSIIHDSKIREHLKLDDDFRPVLHGFHQPADCCFSYTSRKIQCRLMEDYFR
TSSGCPRPGVIFLTKKGQLVCADPSDRRVQDCIRNLTPTSLPEILDTLKLA

>cpoCclN1       blastsearch             scaffold_32
mktlvvgllvllctmmlcscEQDKTHHPSDCCISFVTHQIPKNQVVAYIRPMACASNTVFLTKTRKYICA
NPSDHWVQDYIRKLDRSS

>cpoCcl3a       ENSCPOG00000026400      scaffold_32
meapvaalitliltetlfsQSCCALRGADTPTACCFFFISRKIPRKFVVDYYETSSQCPKAAVIFQTKRG
REVCADLRDTWVHEYIADLELNS

>cpoCcl3b       ENSCPOG00000023103      scaffold_32
mkvsvavlaavlctmtlchqvfsAPIGANTPTACCFTYASKKIPRAYVADYYETSSQCSQPGVIFITKRG
REVCADPNETWVQDYINDLELNA

>cpoCcl3c       ENSCPOG00000019852      scaffold_32
mkvsvavlaavlctmtlchqvfsAPIGADTPTACCFTYASKKIPRAYVADYYETSSQCSQPGVIFITKRG
REVCADPNETWVQDYINDLELNA

>cpoCcl4a       ENSCPOG00000026388      scaffold_32
mkfyvtvlsllvigaafcspvlsAPIGSDPPTACCFTYTQRKLPRNFVTDYYETSSLCSQPAVVFQTKRG
KQVCANPSDSWVQEYVEYLELNEE

>cpoCcl22       ENSCPOG00000004546      scaffold_22
maslqtpllavlillsvmlqatdaGPYGANVEDSICCRDFVRYPLPPRVVKDFYWTSDSCRRAGVVLQTV
KDREICANPKLPWVKKILQKLSP

cpoCx3cl1       ENSCPOG00000004548      scaffold_22
maptplsqllclavlchlivllagQQHGVVKCSNICDKMTSRIPNHLLLGYRLNQASCDKPAVIFETKKH
RFFCADPKEKWVQEARWHLDNRTHALTTQSGGKFEKHVGKPETTLVAQGTSQPALTEPKATEESSSLEPT
ASSQGTQRPTGTSPVLPVRVTGSLGTRLSLPSKAQDGEPPAGTEVFGTTAISTTIARQRSAADQPGSSPG
TQEKAPSTLAPSITPPSTEPTSTQSPTTSHSTTEGQPAWTQGQTPTPESSAGSEMGRGLTHTDAFQDWGP
SSMDHASVVPISSEGTPSQEPVVLGSLIPKAEDPIHGTLDPQRQVLITPVPDTQAATRRQAVGLLAFLGL
```

LFCLGVSMFAYQSLQGCPRKMAGEMVEGLRYVPRSCGSNSYVLVPV

>cpoCcl17 ENSCPOG00000006326 scaffold_22
mtslqmlflaalllgaslqhshaALATNVGRECCLDYFKGAIPIRKLVSWYRTSDECPKDAIVFETVQNR
RICSNPEDRHVKKAVRHLQSRKKSQRATAQD

>cpoCcl27 blastsearch chr1
mkelflptsilmllllllspdpgaaSLLQPITTCCTQLYRRPLPNKILRKVIWVELQEADGDCHLQAFVL
YLTERSVCVHPKNRSLIQWLERHGKRFQGTLRNPNLGL

>cpoCcl19 ENSCPOG00000002627 scaffold_27
marhmapllalsllvlwaslapalgGANDAEDCCLSVTLRPIPGNIVKAFRYLFITDGCRVPAVVFTTLR
GHQLCAPPEQPWVQRIIRRLKKSFAKASRPLPSPIS

>cpoCcl21 ENSCPOG00000005431 scaffold_27
maqmlalrllvlvlalcitqtqgSDGGAQDCCLKYSQKKIPYKVVRSYRKQELSLGCPMPAILFLPKKRS
QPELCADPKEAWVQQLMKRLDNPPVQGCRKDRGNPKAGKRGKGSKGCKRTEKTQGP

>cpoCcl26 ENSCPOG00000000623 scaffold_30
mksshlvfpillafllsghpEAAMRAGNVAEVCCFHYSTKALPVSWVRSYKLTRSSCSCQAVIFTTKRGK
QVCFRPKAKWVQQYIS

>cpoCcl20 ENSCPOG00000010180 scaffold_13
mgcsgkslllaalmsvlllhlcrqseaASSFDCCLRYTRHVLSPKLIVGFTQQLASEACDIDAIIFHTKK
RLSVCADPKKGWVKRAVLVLSQKVKKM

>cpoCcl25 ENSCPOG00000007131 scaffold_195
mnpwllaclvaffvggwvpaMHTQGVSEDCCLAYHPPLRLSVFLHARYFRWQEVSGSCNLPAVIFHFPQP
GRVVCGNPRDKVVRKAMRVLRTQMKSQQQASNVTVQGLQARRNKENSSLSKMHSIRLRHHLGNGKKNAGF
ERDSGSGL

>cpoCcl28 ENSCPOG00000027088 scaffold_29
mqqmrfalvalavcvalqiseaILPIASSCCTEVSHRISRRLLERVTVCRIQRADGDCDLAAVILHVKRR
RICISPHNHTVNQWMKAQAAKKNSKGNICHKKHHSKKKKGSNREKI

>cpoXcl1 ENSCPOG00000012457 scaffold_39
mrllllailgacclaACTVEGVGSEVPESSVCMSLRVHPLPLNRIKTYTIKEGPMRAVIFITKRELKVCA
HPEATWVKKALKSIHSKSSTRRNVTQIHPTGAQQSTYTTVTLPQ

>cpoXcl2 ENSCPOG00000023218 scaffold_39
mrllvlailgacclaACTVEGVGSEVPERSVCVSLTTQRLPVNRIKTYTIKEGSMKAVIFITKRGFKICA
NPEDSWVKAAVRTIGGKSNTR

>itrCxcl8 ENSSTOG00000003271 Scaffold JH393408.1
mtsklvvallvtcllsatlceaAVLTRIGTELRCQCIKTHSTPFHPKYIKELRVIESGPHCANSEIIVKL
VDGRELCLNPKEKWVQKVVELFLKRAENEDP

>itrCxcl6 blast search Scaffold JH393408.1
mslrsslsprlprpsrslcallalllllltppaplanaGPVAAAVVRELRCVCLSTTPGIHPKMVTNLQVI
AAGPQCPKVEVVASLKNGKEVCLDPEAPLIKKIIQKILDGGNKN

>itrCxcl7 ENSSTOG00000023838 Scaffold JH393408.1
mslrpsatsfctntrgvrvlqvllllsllltvlvpsttgQRKRNLGKVESVEPYVELRCICVKTISGIHP
SNIQNLEVIRPGAHCAKVQVIATLKDGREICLDPEAPRVKKIIQKLLESDESAA

```
>itrCxcl4       ENSSTOG00000013032      Scaffold JH393408.1
mglilrscslcphlypgllllgllllsavvtvvrsEDPEEDREFQCMCAKTRYKVKVKDITNLEMIKPGP
LCATTQMIATLKSGSRVCLAGQVSFFKALIGKLMQR

>itrCxcl4L      ENSSTOG00000014918      Scaffold JH393408.1
mglilrscslcpqlypgllllgllllsavvtvargGESKKDGDSHCVCVKTKSKVNAENIFKLEVIKAGP
HCATAQMIATLKSGRKICLDRQVSLFKRLMKKLLDS

>itrCxcl3       blast search            Scaffold JH393408.1
martasavpraprllqvallmlllvaasrraagAPVVSELRCQCLQTVQGIHLKNIQSVKVMSPGPHCAQ
TEVIATLKNGQEACLNPEAPLVKKIIHKMLNNGNSS

>itrCxcl15      blast search            Scaffold JH393408.1
maaessptlllallvlgifadpckaQELRCRCIQTHSDFISPQFIETLQLIPEGVQCSRKEIIVTLKDGQ
LVCLDPEAKWVKTLLEKVQLRPRVLTFTFSHFYFNYRYV

>itrCxcl1       blast search            Scaffold JH393408.1
maraasavpraprllqvalfllllvvacrraagAPVVSELRCQCLQTVQGIHLKNIQSVKVTSPGPHCAQ
TEVIATLKNGQEACLNPEAPLVKKIIDKMLNKSSTN

>itrCxcl2       blast search            Scaffold JH393408.1
..ATLKNGQEACLNPEAPLVKKIIHKMLNKGSN

>itrCxcl9       ENSSTOG00000013732      Scaffold JH393486.1
mkktgipfllgliflvlfgvqgAPIMRNDRCSCISTRQGKIHPKALKDIKQFAPSPSCGKTEIIATMKNG
NEECLNPDSGYVKKVVKMWKEKVSQKKKQKKGGKQKMKTFLKAKRPQRPHQKKTT

>itrCxcl10      ENSSTOG00000026861      Scaffold JH393486.1
mnqsavfifcliflltlsgtqgMPLSRTTRCKCIQISNRPVNPKSLEKLELIPASESCPRVEIIATMKKSG
EKRCLNPESKAIKNLLKALRIERSKRSS

>itrCxcl11      ENSSTOG00000009504      Scaffold JH393486.1
mnvkgmaillavivcatitegFPMFKGGRCLCIGPGVKAVKFTDIEKASVIYPSNSCNKIEVIITLKAHK
GQRCLNPRSKQASFIIKQVERKNFLKYRNK

>itrCxcl13      ENSSTOG00000015287      Scaffold JH393486.1
mrlisaalllmllvssslspvhgVLEAYNTNLKCSCVQGTSDFIHVRQIGRLQIIPPGNGCPIKQIIIRL
KNKTTVCVNPQTSWIKKLIEHLPKKKLFSTQAPAMKKIG

>itrCxcl12      ENSSTOG00000005950      Scaffold JH393427.1
mdtkvvvvlalvlaalclsdgKPVSLSYRCPCRFFESHVARVNVRHLKILNTPNCSLQIVARLKSNNRQV
CIDPKLKWIQEYLEKALNKGRREEKVGKKEKIGKKKRQKKRKAAQKRKN

>itrCxcl14      ENSSTOG00000022730      Scaffold JH393407.1
mrlltaalllllalcvarvdgSKCKCSRKGPKIRYSDVKKLEMKPKYPHCEEKMVIITTKSVSRYRGQE
HCLHPKLQSTKRFIKWYNAWNEKRRVYEE

>itrCxcl16      ENSSTOG00000009339      Scaffold JH393487.1
mrpssgpllpeflfllvllilpgdgNEGSVTGSCPCDERISNNPLSWQIMDHYRKYLRAYRDCPPYIRFE
LIRKSVCGSIKNPSVRELMSCFDRGECGYERGKSRVNQGHLLFPSTPIPEPTELAPPDTSTPAQMHLPPT
QQSTQQFKLPSGVLPLDKELTHLKETTTFTVGYNLGIGFKDGEKQRQKEEMEGTAINKSVMVAVLSLLAI
VFILTGVLLYVLCKRRREQSLQCSPGLHYTPVATVFNA

>itrCxcl17      ENSSTOG00000011599      Scaffold JH393516.1
..VARGHRDQRQASGRRLLKGSQECECKDWFLRASKKKPMTEAGLPKKRCPCDHSTVGVKKTRHRRHHKK
SNKHSKACWQFLQRCQLESFALPL

>itrCcl2        ENSSTOG00000028166  Scaffold JH393348.1: 868,214-870,352
mkvsaallclmltvaafstlvlaQPDAVNSPVTCCYTLTSRKIPMKRLMSYRRVTSSKCPKEAVIFTTVL
```

NKEICADPLQKWVEDYVAKMDQKTEGNQNPTGLGTAAPVNANFTTPEPVANLSATSSPSTNPNTSI

>itrCcl7
mkvsaallclmltvaafcipvlaQPDGINISTCCFETRRRKVSVQKLKSYTRITSSTCPREAVIFQTKLN
KEICADSNQQWVKDAIKHLDKKTQDP

>itrCcl11 ENSSTOG00000012718 Scaffold JH393348.1: 835,859-837,710
mkvsavllclmltaailsmqvlaQPASVPTTCCFTVANKKFSIQRLESYRKITGSKCPQKAVIFKTKQDK
EFCADPKQKWVKDAIKYLDQKLQTPTP

>itrCcl12 ENSSTOG00000023292 Scaffold JH393348.1: 805,250-806,907
mkvsvallclvltaatvssQEQAQPGAPLMPITCCFSTINRRIPIQKLQSYTITSTQCPKVAVIFKTKRG
KMICADPSENWVKDSMTLLNQNSQTPKP

>itrCcl1 ENSSTOG00000007409 Scaffold JH393348.1: 781,687-786,234
mkltsvallclllaatwpqdvdsKSMHVSSSRCCFSFAQKRISQKTIQCYRETSSTCPYQAAIFRLKGGR
ESCALKTERWVQGYLGKVKPCLLV

>itrCcl5 ENSSTOG00000015833 Scaffold JH393300.1
mkvsaaalaviltaaafcipasaSPYASDTTPCCFAFISRPVPRNHIKEYFYTSSKCSNLAVVFVTRRNR
QVCADPEKKWVREYINSLEMS

>itrCcl16 blastsearch Scaffold JH393300.1
mvwsvcvpslslpfissSSFLLPLLSLQRPQTAQRWLDPLRHPAGVNIQASCCVTYYEKTLPQKLVKGYR
EALTCHLPAIIFVTKKNREVCANPNDSWVQDYIKDPQIPLLPPKNSASVKIIRTEKGQP

>itrCcl14 blastsearch Scaffold JH393300.1
matvslllllllsvvleaKVPSSSRPYNPSECCFTYVSQAPPRRRIIDYYETSSECPKSGIVFITKKGHP
ICANPKDEWVQDYIKELEES

>itrCcl15 blastsearch Scaffold JH393300.1
mkvsavalsflilgaalgsqaQVIPDPETRELLEREHTFQPPLVNKVFNSPADCCFSYITRSIRCTVMKT
YHKTSSGCSQPAVIFITNKGQHVCANPNDPSVQECMKKLKHNPSS

>itrCcl23 blastsearch Scaffold JH393300.1
mkfsiaalsllilaaalgsqaRVIHDLEKRQLLRIPIKSPIQHGILQEAADCCFIYTRRSIRCSFMEHYY
KTSSGCPQPAVIFFSRKGQRICADPNDGRVQECMANLNLNS

>itrCcl3a blastsearch Scaffold JH393300.1
mkvlvvalfvllctltlraegESVVHLLLMDENQEPSFCCFAYTSRKIPPKFVAFYYETSSTCSSKGIVF
VTKRGRHVCARPNEAWVQEHIRDLEGSSKKH

>itrCcl3b ENSSTOG00000027899 Scaffold JH393300.1
mevpvavlavlffteals SLTCSASLGADTPTACCFFYISRKIPHKFVDDYYETSSQCSKPAVIFQTKRG
RQVCADPSEAWVRAYITDLELNA

>itrCcl3c ENSSTOG00000027458 Scaffold JH393300.1
..GADSPTACCFSYVARQIQRKFIEDYFETSSQCSQPGVIFLTKRGRQVCADPSENWVQEYITDLELNA

>itrCcl4 ENSSTOG00000013313 Scaffold JH393300.1
mklcgialpllvlvaafcspalsAPMGSDPPTACCFSYTLRKLPRNFVMDYFETSSLCSQPAVVFQTKKG
RQVCANPSESWVQEYMDDLELN

>itrCcl22 ENSSTOG00000010260 Scaffold JH393285.1
maslqapllvalillsvalqatdaGPYGANVEDSICCRDYIRLPLPQRLVKSFYWTSESCRRPGVVLQTL
RDREICANPKLPWVKRIIQKLQE

>itrCx3cl1 ENSSTOG00000015132 Scaffold JH393285.1
maptplawllslatlchltilvagQHHGVTKCNITCNTMTRPIPVNLLIGYWKNPESCGRRAVVLETKRH

```
KYFCADPKEKWVQEAMKHLNRPAQTQNGGTFEKQIGSGPTITPATTEGQSHPAFSEPEATGEGNSLEGTT
SSQEAQRSMGTSPELPVGTTDSSGYRSTPTSKAPEDRPPAGPGTAVASTSVAQQSQTGPGLRVIKAHSET
HSTQVLSTEPPSTQTPDMSYPALEETTVSKGQPTSGKGQTPSPEASPGPEEMSPVSTPRGASQDSGPGEM
THAPVVPVSSEGTPSQEPVASGSWAPKTKEPIHTTSNPQSLSNKAAPNTPDLGPQEATRRQAVGLLAFLG
LLFCLGVAMFAYQSLQGCPRKMAGEMVEGLRYVPRSCGSNSYVLVPV

>itrCcl17        ENSSTOG00000015140      Scaffold JH393285.1
msplktlllaalllgaslqsiraARATNVGRECCMEFFKGAIPIRKLVTWYRTSAECPKDAIVLVTMQGK
SICSDPKDRHVTKAVRFLQRLVKSPGPITQKP

>itrCcl27        ENSSTOG00000002852      Scaffold JH393334.1
mkwlsptssllllllllspdpgaaLLLPPSTTCCTQLYRQPLSKKLLRKVIRVELQEADGDCHLQAFVLH
LARRSVCIHPQNRSLARWFERQGKKFPGKRLPA

>itrCcl19        ENSSTOG00000002862      Scaffold JH393334.1
maprvapllalsllvlwnspapalgGANDAEDCCLSVTQRPIPRNIVKAFRYLLLTDGCRVPAVVFTTLR
GHELCAPPDQPWVDRIIRRLNKSSAKASLSSQPYLQPPNPCSRFCPSHDSAYPSSHRASATAIRP

>itrCcl21        ENSSTOG00000021913      Scaffold JH393334.1
maqtlalsllilvlalcvpwtqgSDGGAQDCCLKYSQRKIPYKVVRGYRKQEASLGCPIPAILFLPQKRS
QPELCGDPKEAWVQQLMKRLDKPPAPRKEGQGCRKDRETQKPGKKRKGSKGCKR

>itrCcl24        ENSSTOG00000004986      Scaffold JH393353.1
magpssiiagflllalcvqcIIPTGSVVTPFSCCFNFTAKKIPQKRVVSYQLTSGSTCVQAGVIFTTTAG
RKVCADPSQLWVQEYKKNLDSKQKQPSAEARVRGVKVRRPRHRGNRTAI

>itrCcl26        blastsearch             Scaffold JH393353.1
mksfplvppillafllsvhtSVLRDSDTEKSCCFQYSHKMIPWNWVQAYQFTQISCPQQGVIFTTKIGER
VCVQPKEKWVQRYISLLEARK

>itrCcl20        blastsearch             Scaffold JH393335.1
mmcsgkslllaalvsvlllhlcreseaASNFDCCLRYTEKVFHHRFLVGFTQQLANEACDIDAVIFYTKR
KLAVCADPKKPWVKTAVRRL..

>itrCcl25        ENSSTOG00000003529      Scaffold JH393398.1
mnpwilaslaacflgawvppihtQGSFEDCCLGYYPKPKLAVLRRASFYRIQEVSGSCNLFAIVFYFRQP
GKMVCGNPRDIRVRKAMRLVSQRSDNRLTIQG..

>itrCcl28        ENSSTOG00000005720      Scaffold JH393290.1
mqqtgltlmtlavcvalrnseaILPIASSCCTEVSHHISRRLLERVNSCRIQRADGDCDLAAVILHIKRR
RICVSPHNHTIKQWIRAQAAKKNGKGKVCQKKHHQRNRKVVHGDKQEARGL

>itrXcl1         ENSSTOG00000014588      Scaffold JH393291.1
mrllllallgicclsactvygvgsEVPEKSVCVSLSTQRLPINRIRTYTIKEGSVKAVIFITRRGLKVCA
NPEAEWVKSAVKSVDSRSTTKRSKLQSSPPTVQPTTTSGTLSR
```

Chemokine domains

has, human
mmu, mouse
rno, rat
ocu, rabbit
cpo, guinea pig
itr, squirrel

>hsaCXCL8
ELRCQCIKTYSKPFHPKFIKELRVIESGPHCANTEIIVKLSDGRELCLDPKENWVQRVVEKF

>hsaCXCL6
ELRCTCLRVTLRVNPKTIGKLQVFPAGPQCSKVEVVASLKNGKQVCLDPEAPFLKKVIQKI

>hsaCXCL4L1
DLQCLCVKTTSQVRPRHITSLEVIKAGPHCPTAQLIATLKNGRKICLDLQALLYKKIIKEH

>hsaCXCL1
ELRCQCLQTLQGIHPKNIQSVNVKSPGPHCAQTEVIATLKNGRKACLNPASPIVKKIIEKM

>hsaCXCL4
DLQCLCVKTTSQVRPRHITSLEVIKAGPHCPTAQLIATLKNGRKICLDLQAPLYKKIIKKL

>hsaCXCL7
ELRCMCIKTTSGIHPKNIQSLEVIGKGTHCNQVEVIATLKDGRKICLDPDAPRIKKIVQKK

>hsaCXCL5
ELRCVCLQTTQGVHPKMISNLQVFAIGPQCSKVEVVASLKNGKEICLDPEAPFLKKVIQKI

>hsaCXCL3
ELRCQCLQTLQGIHLKNIQSVNVRSPGPHCAQTEVIATLKNGKKACLNPASPMVQKIIEKI

>hsaCXCL2
ELRCQCLQTLQGIHLKNIQSVKVKSPGPHCAQTEVIATLKNGQKACLNPASPMVKKIIEKM

>hsaCXCL9
KGRCSCISTNQGTIHLQSLKDLKQFAPSPSCEKIEIIATLKNGVQTCLNPDSADVKELIKKW

>hsaCXCL10
TVRCTCISISNQPVNPRSLEKLEIIPASQFCPRVEIIATMKKKGEKRCLNPESKAIKNLLKAV

>hsaCXCL11
RGRCLCIGPGVKAVKVADIEKASIMYPSNNCDKIEVIITLKENKGQRCLNPKSKQARLIIKKV

>hsaCXCL13
SLRCRCVQESSVFIPRRFIDRIQILPRGNGCPRKEIIVWKKNKSIVCVDPQAEWIQRMMEVL

>hsaCXCL12
SYRCPCRFFESHVARANVKHLKILNTPNCALQIVARLKNNNRQVCIDPKLKWIQEYLEKA

>hsaCXCL14
gSKCKCSRKGPKIRYSDVKKLEMKPKYPHCEEKMVIITTKSVSRYRGQEHCLHPKLQSTKRFIKWY

>hsaCXCL16
TGSCYCGKRISSDSPPSVQFMNRLRKHLRAYHRCLYYTRFQLLSWSVCGGNKDPWVQELMSCL

>hsaCXCL17
GQECECKDWFLRAPRRKFMTVSGLPKKQCPCDHFKGNVKKTRHQRHHRKPNKHSRACQQFLKQCQ

>hsaCCL2
PVTCCYNFTNRKISVQRLASYRRITSSKCPKEAVIFKTIVAKEICADPKQKWVQDSMDHL

>hsaCCL7
STTCCYRFINKKIPKQRLESYRRTTSSHCPREAVIFKTKLDKEICADPTQKWVQDFMKHL

>hsaCCL11
PTTCCFNLANRKIPLQRLESYRRITSGKCPQKAVIFKTKLAKDICADPKKKWVQDSMKYL

>hsaCCL8
PITCCFNVINRKIPIQRLESYTRITNIQCPKEAVIFKTKRGKEVCADPKERWVRDSMKHL

>hsaCCL13
PSTCCFTFSSKKISLQRLKSYVITTSRCPQKAVIFRTKLGKEICADPKEKWVQNYMKHL

>hsaCCL1
FSRCCFSFAEQEIPLRAILCYRNTSSICSNEGLIFKLKRGKEACALDTVGWVQRHRKML

>hsaCCL5
TTPCCFAYIARPLPRAHIKEYFYTSGKCSNPAVVFVTRKNRQVCANPEKKWVREYINSL

>hsaCCL16
PSTCCLKYYEKVLPRRLVVGYRKALNCHLPAIIFVTKRNREVCTNPNDDWVQEYIKDP

>hsaCCL14
PSECCFTYTTYKIPRQRIMDYYETNSQCSKPGIVFITKRGHSVCTNPSDKWVQDYIKDM

>hsaCCL15
AADCCTSYISQSIPCSLMKSYFETSSECSKPGVIFLTKKGRQVCAKPSGPGVQDCMKKL

>hsaCCL23
SADCCISYTPRSIPCSLLESYFETNSECSKPGVIFLTKKGRRFCANPSDKQVQVCVRML

>hsaCCL18
KELCCLVYTSWQIPQKFIVDYSETSPQCPKPGVILLTKRGRQICADPNKKWVQKYISDL

>hsaCCL3
PTACCFSYTSRQIPQNFIADYFETSSQCSKPGVIFLTKRSRQVCADPSEEWVQKYVSDL

>hsaCCL4
PTACCFSYTARKLPRNFVVDYYETSSLCSQPAVVFQTKRSKQVCADPSESWVQEYVYDL

>hsaCCL3L3
PTACCFSYTSRQIPQNFIADYFETSSQCSKPSVIFLTKRGRQVCADPSEEWVQKYVSDL

>hsaCCL4L1
PTACCFSYTARKLPRNFVVDYYETSSLCSQPAVVFQTKRGKQVCADPSESWVQEYVYDL

>hsaCCL3L1
PTACCFSYTSRQIPQNFIADYFETSSQCSKPSVIFLTKRGRQVCADPSEEWVQKYVSDL

>hsaCCL4L2
PTACCFSYTARKLPRNFVVDYYETSSLCSQPAVVFQTKRGKQVCADPSESWVQEYVYDL

>hsaCCL22
DSVCCRDYVRYRLPLRVVKHFYWTSDSCPRPGVVLLTFRDKEICADPRVPWVKMILNKL

>hsaCX3CL1
VTKCNITCSKMTSKIPVALLIHYQQNQASCGKRAIILETRQHRLFCADPKEQWVKDAMQHL

>hsaCCL17
GRECCLEYFKGAIPLRKLKTWYQTSEDCSRDAIVFVTVQGRAICSDPNNKRVKNAVKYL

>hsaCCL27
STACCTQLYRKPLSDKLLRKVIQVELQEADGDCHLQAFVLHLAQRSICIHPQNPSLSQWFEHQ

>hsaCCL19
AEDCCLSVTQKPIPGYIVRNFHYLLIKDGCRVPAVVFTTLRGRQLCAPPDQPWVERIIQRL

>hsaCCL21
AQDCCLKYSQRKIPAKVVRSYRKQEPSLGCSIPAILFLPRKRSQAELCADPKELWVQQLMQHL

>hsaCCL26
SKTCCFQYSHKPLPWTWVRSYEFTSNSCSQRAVIFTTKRGKKVCTHPRKKWVQKYISLL

>hsaCCL24
PSPCCMFFVSKRIPENRVVSYQLSSRSTCLKAGVIFTTKKGQQFCGDPKQEWVQRYMKNL

>hsaCCL20
NFDCCLGYTDRILHPKFIVGFTRQLANEGCDINAIIFHTKKKLSVCANPKQTWVKYIVRLL

>hsaCCL25
FEDCCLAYHYPIGWAVLRRAWTYRIQEVSGSCNLPAAIFYLPKRHRKVCGNPKSREVQRAMKLL

>hsaCCL28
ASSCCTEVSHHISRRLLERVNMCRIQRADGDCDLAAVILHVKRRRICVSPHNHTVKQWMKVQ

>hsaXCL2
RRTCVSLTTQRLPVSRIKTYTITEGSLRAVIFITKRGLKVCADPQATWVRDVVRSM

>hsaXCL1
KRTCVSLTTQRLPVSRIKTYTITEGSLRAVIFITKRGLKVCADPQATWVRDVVRSM

>mmuCxcl5
ELRCVCLTVTPKINPKLIANLEVIPAGPQCPTVEVIAKLKNQKEVCLDPEAPVIKKIIQKI

>mmuCxcl7
ELRCRCTNTISGIPFNSISLVNVYRPGVHCADVEVIATLKNGQKTCLDPNAPGVKRIVMKI

>mmuCxcl4
DLSCVCVKTISSGIHLKHITSLEVIKAGRHCAVPQLIATLKNGRKICLDRQAPLYKKVIKKI

>mmuCxcl3
ELRCQCLNTLPRVDFETIQSLTVTPPGPHCTQTEVIATLKDGQEVCLNPQGPRLQIIIKKI

>mmuCxcl15
ELRCLCIQEHSEFIPLKLIKNIMVIFETIYCNRKEVIAVPKNGSMICLDPDAPWVKATVGPI

>mmuCxcl1
ELRCQCLQTMAGIHLKNIQSLKVLPSGPHCTQTEVIATLKNGREACLDPEAPLVQKIVQKM

>mmuCxcl2
ELRCQCLKTLPRVDFKNIQSLSVTPPGPHCAQTEVIATLKGGQKVCLDPEAPLVQKIIQKI

>mmuCxcl9
NARCSCISTSRGTIHYKSLKDLKQFAPSPNCNKTEIIATLKNGDQTCLDPDSANVKKLMKEW

>mmuCxcl10
TVRCNCIHIDDGPVRMRAIGKLEIIPASLSCPRVEIIATMKKNDEQRCLNPESKTIKNLMKAF

>mmuCxcl11
QGRCLCIGPGMKAVKMAEIEKASVIYPSNGCDKVEVMCLDPRSKQARLIMQAI

>mmuCxcl13
NLKCRCSGVISTVVGLNIIDRIQVTPPGNGCPKTEVVIWTKMKKVICVNPRAKWLQRLLRHV

>mmuCxcl12
SYRCPCRFFESHIARANVKHLKILNTPNCALQIVARLKNNNRQVCIDPKLKWIQEYLEKA

>mmuCxcl14
gSKCKCSRKGPKIRYSDVKKLEMKPKYPHCEEKMVIVTTKSMSRYRGQEHCLHPKLQSTKRFIKWY

>mmuCxcl16
AGSCSCDRTISSGTQIPQGTLDHIRKYLKAFHRCPFFIRFQLQSKSVCGGSQDQWVRELVDCF

>mmuCxcl17
GQECECKDWFLQAPKRKATAVLGPPRKQCPCDHVKGREKKNRHQKHHRKSQRPSRACQQFLKRCH

>mmuCcl2
PLTCCYSFTSKMIPMSRLESYKRITSSRCPKEAVVFVTKLKREVCADPKKEWVQTYIKNL

>mmuCcl7
ASTCCYVKKQKIPKRNLKSYRRITSSRCPWEAVIFKTKKGMEVCAEAHQKWVEEAIAYL

>mmuCcl11
PTSCCFIMTSKKIPNTLLKSYKRITNNRCTLKAIVFKTRLGKEICADPKKKWVQDATKHL

>mmuCcl12
PVTCCYNVVKQKIHVRKLKSYRRITSSQCPREAVIFRTILDKEICADPKEKWVKNSINHL

>mmuCcl8
PVTCCFHVLKLKIPLRVLKSYERINNIQCPMEAVVFQTKQGMSLCVDPTQKWVSEYMEIL

>mmuCcl1
SNSCCLNTLKKELPLKFIQCYRKMGSSCPDPPAVVFRLNKGRESCASTNKTWVQNHLKKV

>mmuCcl5
TTPCCFAYLSLALPRAHVKEYFYTSSKCSNLAVVFVTRRNRQVCANPEKKWVQEYINYL

>mmuCcl9
SSDCCLSYNSRIQCSRFIGYFPTSGGCTRPGIIFISKRGFQVCANPSDRRVQRCIERL

>mmuCcl6
SSDCCFSYATQIPCKRFIYYFPTSGGCIKPGIIFISRRGTQVCADPSDRRVQRCLSTL

>mmuCcl3
PTACCFSYSRKIPRQFIVDYFETSSLCSQPGVIFLTKRNRQICADSKETWVQEYITDL

>mmuCcl4
PTSCCFSYTSRQLHRSFVMDYYETSSLCSKPAVVFLTKRGRQICANPSEPWVTEYMSDL

>mmuCcl22
DSICCQDYIRHPLPSRLVKEFFWTSKSCRKPGVVLITVKNRDICADPRQVWVKKLLHKL

>mmuCx3cl1
MTKCEIMCDKMTSRIPVALLIRYQLNQESCGKRAIVLETTQHRRFCADPKEKWVQDAMKHL

>mmuCcl17
GRECCLDYFKGAIPIRKLVSWYKTSVECSRDAIVFLTVQGKLICADPKDKHVKKAIRLV

>mmuCcl27a
STSCCTQLYRQPLPSRLLRRIVHMELQEADGDCHLQAVVLHLARRSVCVHPQNRSLARWLERQ

>mmuCcl21b
GQDCCLKYSQKKIPYSIVRGYRKQEPSLGCPIPAILFLPRKHSKPELCANPEEGWVQNLMRRL

>mmuCcl19b
AEDCCLSVTQRPIPGNIVKAFRYLLNEDGCRVPAVVFTTLRGYQLCAPPDQLWVDRIIRRL

>mmuCcl21d
GQDCCLKYSQKKIPYSIVRGYRKQEPSLGCPIPAILFLPRKHSKPELCANPEEGWVQNLMRRL

>mmuCcl27c
STSCCTQLYRQPLPSRLLRRIVHMELQEADGDCHLQAVVLHLARRSVCVHPQNRSLARWLERQ

>mmuCcl21c
GQDCCLKYSQKKIPYSIVRGYRKQEPSLGCPIPAILFLPRKHSKPELCANPEEGWVQNLMRRL

>mmuCcl19c
AEDCCLSVTQRPIPGNIVKAFRYLLNEDGCRVPAVVFTTLRGYQLCAPPDQPWVDRIIRRL

>mmuCcl27b
STSCCTQLYRQPLPSRLLRRIVHMELQEADGDCHLQAVVLHLARRSVCVHPQNRSLARWLERQ

>mmuCcl21e
GQDCCLKYSQKKIPYSIVRGYRKQEPSLGCPIPAILFLPRKHSKPELCANPEEGWVQNLMRRL

>mmuCcl19
AEDCCLSVTQRPIPGNIVKAFRYLLNEDGCRVPAVVFTTLRGYQLCAPPDQPWVDRIIRRL

>mmuCcl21a
GQDCCLKYSQKKIPYSIVRGYRKQEPSLGCPIPAILFSPRKHSKPELCANPEEGWVQNLMRRL

>mmuCcl26
AMSCCPNFSYYVIPWSWVYSYKFTDKSCTSDGVIFFTKTGKQFCVQPGAKWVQRFISLV

>mmuCcl24
PSSCCTSFISKKIPENRVVSYQLANGSICPKAGVIFITKKGHKICTDPKLLWVQRHIQKL

>mmuCcl20
NYDCCLSYIQTPLPSRAIVGFTRQMADEACDINAIIFHTKKRKSVCADPKQNWVKRAVNLL

>mmuCcl25
FEDCCLGYQHRIKWNVLRHARNYHQQEVSGSCNLRAVRFYFRQKVVCGNPEDMNVKRAMRIL

>mmuCcl28
ASSCCTEVSHHVSGRLLERVSSCSIQRADGDCDLAAVILHVKRRRICISPHNRTLKQWMRAS

>mmuXcl1

ESSCVNLQTQRLPVQKIKTYIIWEGAMRAVIFVTKRGLKICADPEAKWVKAAIKTV

>rnoCxcl6
ELRCVCLTLAPRINPKMIANLEVIPAGPHCPKVEVIAKLKNQKDNVCLDPEAPLIKKVIQKI

>rnoCxcl7
ELRCRCTNTLSGIPLNSISRVNVFRPGAHCDNVEVIATLKNGKEVCLDPTAPMIKKIVKKI

>rnoCxcl4
DLSCVCVKTSSSRIHLKRITSLEVIKAGPHCAVPQLIATLKNGSKICLDRQVPLYKKIIKKL

>rnoCxcl3
ELRCQCLKTLPRVDFENIQSLTVTPPGPHCTQTEVIATLKDGQEVCLNPQAPRLQKIIQKL

>rnoCxcl15
ELRCQCIQITSESIPCKFIKTIEVIYENIYCNRMEVIAVLRDGNAICLHPNSTCVRTLIDNI

>rnoCxcl1
ELRCQCLQTVAGIHFKNIQSLKVMPPGPHCTQTEVIATLKNGREACLDPEAPMVQKIVQKM

>rnoCxcl2
ELRCQCLTTLPRVDFKNIQSLTVTPPGPHCAQTEVIATLKDGHEVCLNPEAPLVQRIVQKI

>rnoCxcl9
NQRCSCISTSQGTFHYKSLKDLKQFAPSPNCNKTEIIATLKNGDQTCLDPDSARVKKLMKEW

>rnoCxcl10
TVRCTCIDFHEQPLRPRAIGKLEIIPASLSCPHVEIIATMKKNNEKRCLNPESEAIKSLLKAV

>rnoCxcl11
GGRCLCIDRGVKVVKMAAIKEVSVIYPSNGCDKVEVIVTLKAHKGQRCLDPTSKQARLIMQTI

>rnoCxcl13
NLKCRCSKVSSTFINLILVDWIQVIRPGNGCPKTEIIFWTKAKKAICVNPTARWLPKVLKFV

>rnoCxcl12
SYRCPCRFFESHVARANVKHLKILNTPNCALQIVARLKSNNRQVCIDPKLKWIQEYLDKA

>rnoCxcl14
gSKCKCSRKGPKIRYSDVKKLEMKPKYPHCEEKMVIITTKSMSRYRGQEHCLHPKLQSTKRFIKWY

>rnoCxcl16
AGSCYCDRTIPSGTQIPPATLNHMRKYLKTFHRCQFFIRFQLQSKSVCGGSQDQWVRELVNCF

>rnoCxcl17
GQECDCKDWSLRVSKRKTTAVLEPPRKQCPCDHVKGSEKKNRRQKHHRKSQRPSRTCQQFLKRCQ

>rnoCcl2
PLTCCYSFTGKMIPMSRLENYKRITSSRCPKEAVVFVTKLKREICADPNKEWVQKYIRKL

>rnoCcl7
SSTCCYVKKQKIPKRNLKSYRKITSSRCPWEAVIFKTKKGMEVCAEAHQKWVEEAIAYL

>rnoCcl11
PTSCCFTMTSKKIPNTLLKSYKRITNNRCTLKAIVFKTKLGKEICADPKKKWVQDATKHL

```
>rnoCcl12
PVTCCYNVAKQKIHIRRLKSYRKITSSQCPREAVIFRTVLDKELCADPKEKWVKDSMNHL

>rnoCcl1
SSRCCLNTLENKIALKFIKCYKEIGPSCPYYPAVIFRLIKGRESCALTNTTWVQDYLKKV

>rnoCcl5
TTPCCFAYLSLALPRAHVKEYFYTSSKCSNLAVVFVTRRNRQVCANPEKKWVQEYINYL

>rnoCcl9
SSDCCLSYTSRVHCSRYKNYFPTSGGCTKPAIIFVSKKGTLVCANPSDLRVQKCIKRL

>rnoCcl6
SSDCCFSYASQIPCSRFIYYFPTSGGCTKPGIIFVTRKRKRVCANPSDQRVQTCISTL

>rnoCcl3
PTACCFSYGRQIPRKFIADYFETSSLCSQPGVIFLTKRNRQICADPKETWVQEYITEL

>rnoCcl4
PTSCCFSYTSRKIHRNFVMDYYETSSLCSQPAVVFLTKKGRQICADPSEPWVNEYVNDL

>rnoCcl22
DSICCQDYIRHPLPPRFVKEFYWTSKSCRKPGVVLITIKNRDICADPRMLWVKKILHKL

>rnoCx3cl1
MTKCNITCHKMTSPIPVTLLIHYQLNQESCGKRAIILETRQHRHFCADPKEKWVQDAMKHL

>rnoCcl17
GRECCLDYFKGAIPIRKLVTWFRTSVECPRDAIVFETVQGRLICTDPKDKHVKKAIRHL

>rnoCcl27
STSCCTQLYRQPLPTRLLRRIVRVELQEADGDCHLQAVVLHLTRRSVCVHPQNRSLARWLERH

rnoCcl19
AEDCCLSVTQRPIPGNIVKAFRYLLIQDGCRVPAVVFTTLRGYQLCAPLDQPWVERIIRRL

>rnoCcl21
GQDCCLRYSQKKIPYNIVRGYRKQEPSLGCPIPAILFAPRKQSQPELCANPEEAWVQKLMRRL

>rnoCcl26
PMPCCSYFSHYVIPWHWLYSYQITDSSCPNEGVIFTTKTGKQYCVQPGDKWVQRYISLL

>rnoCcl24
PSSCCVTFISKKIPVNRVISYQLANGSICPKAGVIFITKKGHKICTDPKLPWVQKHIKNL

>rnoCcl20
NFDCCLTYTKNVYHHARNFVGFTTQMADEACDINAIIFHLKSKRSVCADPKQIWVKRILHLL

>rnoCcl25
FEDCCLGYQPRIKWNVLRHARHYHQQEVSGSCNLRAVIFYFRQKVVVCGNPEDKDVKRAVKIL

>rnoCcl28
ASSCCTEVSHHIPRRLLERVNSCSIQRADGDCDLAAVILHVKRRRICVSPHNPTLKRWMSAS

>rnoXcl1
ESICVSLRTQRLPVQKIKTYTIKEGAMRAVIFVTKRGLRICADPQAKWVKTAIKTV
```

>ocuCxcl8
ELRCQCIKTHSTPFHPKFIKELRVIESGPHCANSEIIVKLVDGRELCLDPKEKWVQKVVQIF

>ocuCxcl6
ELRCVCLSTTAIHPKMIARLQVIAAGPQCSKVEVVASLKNGKEICLDPEAPLIKKAIQKI

>ocuCxcl4
DLHCVCVKTTSLVRPRHITNLELIKAGAHCPTAQLITLKNGRKLCLDQAALYKKVIKKLL

>ocuCxcl3
ELRCQCLQTVQGIHLKNIQNLKVLSPGPHCAQTEVIATLKSGQEACLNPAAPMVKKFLQKR

>ocuCxcl15
ELRCQCIQIYSNFISPKLIKNVQMIPSGPYCSTKEVIVTLKDGRLICLDPEAEWVMTLIKNT

>ocuCxcl1
ELRCQCLQTVQGIHLKSIQSLKVLSPGPHCAQTEVIATLKSGQEACLNPAAPMVKKFLQKR

>ocuCxcl2
ELRCQCLQTVQGIHLKSIQSLKVLSPGPHCAQTEVIATLKSGQEACLNPASPMVKKLLQKM

>ocuCXCL9
NGRCSCISSTQGKIHLQSLKDLKQFSPSPSCGKTEIIATKKDGTQICLNPDSTEVKELVEKW

>ocuCXCL10
TVRCTCINISNKPVNPRSLEKLEIIPASQSCANVEIIATMKKDGEKRCLNPELKAIKKLLKAF

>ocuCXCL11
GGRCLCTGPGVKAVKVADVQKATIIYPSGSCDKTEVIITLKAKKGQQCLNPRSKQASLIIKQI

>ocuCXCL13
DLKCECVQETANYIPIRFIDRLQIVPPGNGCPKREVIVWLKTKIAVCVKPHAKWLQKLIQIL

>ocuCXCL16
TGSCHCDQIISSSSPPNPKLMEHFRKHLRAYHRCTAYIRFRLHSRSVCGGNHDPWVHELLRCF

>ocuCXCL17
GQECECKGWFLRAPKRKLMTVPRLPKKHCPCDHFKGTVKKTRHQKSHRQPNKHSRACQQFLKRCQ

>ocuCcl2
PVTCCYTFTSKTISVKRLMSYRRINSTKCPKEAVIFMTKLAKGICADPKQKWVQDAIANL

>ocuCcl7
GKTCCYRFHNRRMDPQKLRSYTLISISYCPREAVIFKTKQHREVCADPKWPWAQNAIAYL

>ocuCcl11
PTTCCFSMAKKMPLQRLESYRRISGSKCPQKAVIFKTKLAKEICADPKEKWVQDSIKYL

>ocuCcl13
LTACCFSFVRKRIPLQRLVSYRKTSKACVKEAVIFRTRRDQELCADPMQKWVQDTMRVL

>ocuCcl1
SSNCCFSFVEKRIPLKTIHCYRRSSSTCPYDAIIFKLRGGRESCALKTVGWLQGYFKKM

>ocuCCL5
TTPCCFAYISRLLPRAHVTEYFYTSGKCSFPAVVFVTRKNRQVCANPEKKWVREYINSL

```
>ocuCCL14
PAECCFSYITRVVPRQRITDYYETSSECSKPGIVFITKKGYSICANPRDDWVQDYIRDL

>ocuCCL15
PSDCCYGYTTRPLQCSVMEDFFETNSQCSMPAVIFITKCGKLICANPRNNEVEECMETL

>ocuCCL23
PTDCCFSYTKRNIQCFLMQDYIETSSQCSMPAVIFLTKRGQKVCADSSDERVRKCVSSL

>ocuCCL3
PTACCFSYISRQIPYKFIADYFETSSQCSKPGVIFLTKRGRQVCADISEAWVQGYINDL

>ocuCCL4
PTACCFSYTLRQLPRHFVIDYFETSSLCSQPAVVFQTKKGRQVCANPSESWVQEYVDDL

>ocuCCL22
DSVCCRDYMRRPLAPRVVKYFYWTSDSCRRPGVVLVTLKGLEICADPRLPWVKKILRKL

>ocuCX3CL1
VAKCSIICNNMTPKIPVALLISYQWNQESCGKPAIVLETKRHKRFCADPQAPWVQEAMKHL

>ocuCCL27
STTCCTQLYRQPLSNKLLRKVIRVELQEADGDCHLQAFVLHLARRSVCIHPQNRSLARWFERQ

>ocuCCL19
AEDCCLSVTQRPIPSNIVRAFRYLLVTDGCRVPAVVFTTLRGFQLCAPPEQPWVGRIIRRL

>ocuCCL21
AQDCCLKYSLRKIPHKVVRSYRKQEPSLGCPISAILFSPRKRSQPELCADPKEPWVQQLMKRL

>ocuCCL24
PSSCCMNFIPKKIPESQVVSYQLSSGSVCPRAGVIFTTKKGYRVCGDPKQQWVQSYVRNL

>ocuCCL20
NFDCCLRYTECRIHPRFIVGFTQQLANEACDINAIILHTKKNLSVCADPKKEWVKRVVHIL

>ocuCCL25
SEDCCLAYHSLSWAVLQRTQGYQLQEVSGSCNLRAVRFCLKHRIVCGNPQDKKVQRAVKLL

>ocuCCL28
FSSCCTEVSPHLSRRLLKMVHTCHLQRADGDCDLDAVALYFGRRRFCVTTENHMLKEWMKAQ

>ocuXCL1
KSICVSLTTQPLPVNRIRTYTIKEGPMKAVIFITKRGLKVCADPQAKWVKTAIKSI



>cpoCxcl8
ELRCQCIKIHTTPFHPKFIKELKVIESGPRCANSEIIVKLSDNRQLCLDPKKKWVQDVVSMF

>cpoCxcl6
ELRCVCVTITPGVHPKMIASLQVTVSGPQCPNVEVVATLKNGKQICLDPEAPLIKRVIQKM

>cpoCxcl7
ELRCVCLSTTAIHPKMIARLQVIAAGPQCSKVEVVASLKNGKEICLDPEAPLIKKAIQKI
```

>cpoCxcl1
ELRCRCLRPVRGLHPKNIQSVAVTAPGPHCHQTEVLATLKDGREACLDPEAPMVQKVLQRM

>cpoCxcl9
KGRCYCINSSDDVISLKSLKDLKQFVPSPSCEKTEIIATKRNGDQTCLNPDSTKVKKLIKQW

>cpoCxcl10
TIRCTCIETSTQPVNPKSFKKLEIIPASQSCPRVEIIATMKMNGEKRCLDPESKVIKNLLKAV

>cpoCxcl11
RGRCLCIGPGLRAVKVADIAKASIIYPSNSCDKIEVIITLKAHKGQRCLNPRSKQAGLIIKQV

>cpoCxcl13
NLKCRCMQETSQVVRIPNIHRLRILPPGNGCPKTQIIIQLRNKTLVCLNPAAKWVQQFVARV

>cpoCxcl12
SYRCPCRFFESHVARANVKHLKILNTPNCALQIVARLKNNNRQVCIDPKLKWIQEYLEKA

>cpoCxcl17
SRECECKDWFQRALRRKPMTVPALPKKQCPCDHLKVNMKKSRHHKHQRKPNKHSRACQEFLKQCQ

>cpoCcl2L
TPICCYVFNRKIPLKRVRGYERITSSRCPQEAVIFRTLKNKEVCADPTQKWVQDYIAKL

>cpoCcl2
TPTCCYTFNKQIPLKRVKGYERITSSRCPQEAVIFRTLKNKEVCADPTQKWVQDYIAKL

>cpoCcl7
ISTCCYKRSQRIRVQRLESYTRITSSKCPWQAVIFKTKFNREICADPKQQWVQDSMKYI

>cpoCcl11
PSACCFRVTNKKISFQRLKSYKIITSSKCPQTAIVFEIKPDKMICADPKKKWVQDAKKYL

>cpoCcl13
PLACCLTLTHRKIPLNYLKGYELTSDKCPLTAVIFKTRRNIQICADPTKKWVQHAMVYL

>cpoCcl1
SSRCCYSFVERKISHHRIQSYKGTSSSCPYEAVIFKLKGGREICALKTIEWVQDYLTKK

>cpoCcl5
TTPCCFAYISRALPRTHIKEYFYTSSKCSNLAVVFVTRKNRQVCANPEKKWVREYINSL

>cpoCcl14
PSECCFSYITHAVLPYRIMGYYETNGDCPKPAVVFITTKGHRICANPENEWVQDYLKDL

>cpoCcl15
PADCCFSYTSRKIQCRLMEDYFRTSSGCPRPGVIFLTKKGKLVCADPSDRRVQDCIRNL

>cpoCcl23
PADCCFSYTSRKIQCRLMEDYFRTSSGCPRPGVIFLTKKGQLVCADPSDRRVQDCIRNL

>cpoCclN1
PSDCCISFVTHQIPKNQVVAYIRPMACASNTVFLTKTRKYICANPSDHWVQDYIRKL

>cpoCcl3a
PTACCFFFISRKIPRKFVVDYYETSSQCPKAAVIFQTKRGREVCADLRDTWVHEYIADL

>cpoCcl3b
PTACCFTYASKKIPRAYVADYYETSSQCSQPGVIFITKRGREVCADPNETWVQDYINDL

```
>cpoCcl3c
PTACCFTYASKKIPRAYVADYYETSSQCSQPGVIFITKRGREVCADPNETWVQDYINDL

>cpoCcl4a
PTACCFTYTQRKLPRNFVTDYYETSSLCSQPAVVFQTKRGKQVCANPSDSWVQEYVEYL

>cpoCcl22
DSICCRDFVRYPLPPRVVKDFYWTSDSCRRAGVVLQTVKDREICANPKLPWVKKILQKL

cpoCx3cl1
VVKCSNICDKMTSRIPNHLLLGYRLNQASCDKPAVIFETKKHRFFCADPKEKWVQEARWHL

>cpoCcl17
GRECCLDYFKGAIPIRKLVSWYRTSDECPKDAIVFETVQNRRICSNPEDRHVKKAVRHL

>cpoCcl27
ITTCCTQLYRRPLPNKILRKVIWVELQEADGDCHLQAFVLYLTERSVCVHPKNRSLIQWLERH

>cpoCcl19
AEDCCLSVTLRPIPGNIVKAFRYLFITDGCRVPAVVFTTLRGHQLCAPPEQPWVQRIIRRL

>cpoCcl21
AQDCCLKYSQKKIPYKVVRSYRKQELSLGCPMPAILFLPKKRSQPELCADPKEAWVQQLMKRL

>cpoCcl26
AEVCCFHYSTKALPVSWVRSYKLTRSSCSCQAVIFTTKRGKQVCFRPKAKWVQQYIS

>cpoCcl20
SFDCCLRYTRHVLSPKLIVGFTQQLASEACDIDAIIFHTKKRLSVCADPKKGWVKRAVLVL

>cpoCcl25
SEDCCLAYHPPLRLSVFLHARYFRWQEVSGSCNLPAVIFHFPQPGRVVCGNPRDKVVRKAMRVL

>cpoCcl28
ASSCCTEVSHRISRRLLERVTVCRIQRADGDCDLAAVILHVKRRRICISPHNHTVNQWMKAQ

>cpoXcl1
SSVCMSLRVHPLPLNRIKTYTIKEGPMRAVIFITKRELKVCAHPEATWVKKALKSI

>cpoXcl2
RSVCVSLTTQRLPVNRIKTYTIKEGSMKAVIFITKRGFKICANPEDSWVKAAVRTI


>itrCxcl8
ELRCQCIKTHSTPFHPKYIKELRVIESGPHCANSEIIVKLVDGRELCLNPKEKWVQKVVELF

>itrCxcl6
ELRCVCLSTTPGIHPKMVTNLQVIAAGPQCPKVEVVASLKNGKEVCLDPEAPLIKKIIQKI

>itrCxcl7
ELRCICVKTISGIHPSNIQNLEVIRPGAHCAKVQVIATLKDGREICLDPEAPRVKKIIQKL

>itrCxcl4
EFQCMCAKTRYKVKVKDITNLEMIKPGPLCATTQMIATLKSGSRVCLAGQVSFFKALIGKL
```

>itrCxcl4L
DSHCVCVKTKSKVNAENIFKLEVIKAGPHCATAQMIATLKSGRKICLDRQVSLFKRLMKKL

>itrCxcl3
ELRCQCLQTVQGIHLKNIQSVKVMSPGPHCAQTEVIATLKNGQEACLNPEAPLVKKIIHKM

>itrCxcl15
ELRCRCIQTHSDFISPQFIETLQLIPEGVQCSRKEIIVTLKDGQLVCLDPEAKWVKTLLEKV

>itrCxcl1
ELRCQCLQTVQGIHLKNIQSVKVTSPGPHCAQTEVIATLKNGQEACLNPEAPLVKKIIDKM

>itrCxcl9
NDRCSCISTRQGKIHPKALKDIKQFAPSPSCGKTEIIATMKNGNEECLNPDSGYVKKVVKMW

>itrCxcl10
TTRCKCIQISNRPVNPKSLEKLELIPASESCPRVEIIATMKKSGEKRCLNPESKAIKNLLKAL

>itrCxcl11
GGRCLCIGPGVKAVKFTDIEKASVIYPSNSCNKIEVIITLKAHKGQRCLNPRSKQASFIIKQV

>itrCxcl13
NLKCSCVQGTSDFIHVRQIGRLQIIPPGNGCPIKQIIIRLKNKTTVCVNPQTSWIKKLIEHL

>itrCxcl12
SYRCPCRFFESHVARVNVRHLKILNTPNCSLQIVARLKSNNRQVCIDPKLKWIQEYLEKA

>itrCxcl14
gSKCKCSRKGPKIRYSDVKKLEMKPKYPHCEEKMVIITTKSVSRYRGQEHCLHPKLQSTKRFIKWY

>itrCxcl16
TGSCPCDERISNNPLSWQIMDHYRKYLRAYRDCPPYIRFELIRKSVCGSIKNPSVRELMSCF

>itrCxcl17
SQECECKDWFLRASKKKPMTEAGLPKKRCPCDHSTVGVKKTRHRRHHKKSNKHSKACWQFLQRCQ

>itrCcl2
PVTCCYTLTSRKIPMKRLMSYRRVTSSKCPKEAVIFTTVLNKEICADPLQKWVEDYVAKM

>itrCcl7
ISTCCFETRRRKVSVQKLKSYTRITSSTCPREAVIFQTKLNKEICADSNQQWVKDAIKHL

>itrCcl11
PTTCCFTVANKKFSIQRLESYRKITGSKCPQKAVIFKTKQDKEFCADPKQKWVKDAIKYL

>itrCcl12
PITCCFSTINRRIPIQKLQSYTITSTQCPKVAVIFKTKRGKMICADPSENWVKDSMTLL

>itrCcl1
SSRCCFSFAQKRISQKTIQCYRETSSTCPYQAAIFRLKGGRESCALKTERWVQGYLGKV

>itrCcl5
TTPCCFAFISRPVPRNHIKEYFYTSSKCSNLAVVFVTRRNRQVCADPEKKWVREYINSL

>itrCcl16
QASCCVTYYEKTLPQKLVKGYREALTCHLPAIIFVTKKNREVCANPNDSWVQDYIKDP

>itrCcl14
PSECCFTYVSQAPPRRRIIDYYETSSECPKSGIVFITKKGHPICANPKDEWVQDYIKEL

>itrCcl15
PADCCFSYITRSIRCTVMKTYHKTSSGCSQPAVIFITNKGQHVCANPNDPSVQECMKKL

>itrCcl23
AADCCFIYTRRSIRCSFMEHYYKTSSGCPQPAVIFFSRKGQRICADPNDGRVQECMANL

>itrCcl3a
PSFCCFAYTSRKIPPKFVAFYYETSSTCSSKGIVFVTKRGRHVCARPNEAWVQEHIRDL

>itrCcl3b
PTACCFFYISRKIPHKFVDDYYETSSQCSKPAVIFQTKRGRQVCADPSEAWVRAYITDL

>itrCcl3c
PTACCFSYVARQIQRKFIEDYFETSSQCSQPGVIFLTKRGRQVCADPSENWVQEYITDL

>itrCcl4
PTACCFSYTLRKLPRNFVMDYFETSSLCSQPAVVFQTKKGRQVCANPSESWVQEYMDDL

>itrCcl22
DSICCRDYIRLPLPQRLVKSFYWTSESCRRPGVVLQTLRDREICANPKLPWVKRIIQKL

>itrCx3cl1
VTKCNITCNTMTRPIPVNLLIGYWKNPESCGRRAVVLETKRHKYFCADPKEKWVQEAMKHL

>itrCcl17
GRECCMEFFKGAIPIRKLVTWYRTSAECPKDAIVLVTMQGKSICSDPKDRHVTKAVRFL

>itrCcl27
STTCCTQLYRQPLSKKLLRKVIRVELQEADGDCHLQAFVLHLARRSVCIHPQNRSLARWFERQ

>itrCcl19
AEDCCLSVTQRPIPRNIVKAFRYLLLTDGCRVPAVVFTTLRGHELCAPPDQPWVDRIIRRL

>itrCcl21
AQDCCLKYSQRKIPYKVVRGYRKQEASLGCPIPAILFLPQKRSQPELCGDPKEAWVQQLMKRL

>itrCcl24
PFSCCFNFTAKKIPQKRVVSYQLTSGSTCVQAGVIFTTTAGRKVCADPSQLWVQEYKKNL

>itrCcl26
EKSCCFQYSHKMIPWNWVQAYQFTQISCPQQGVIFTTKIGERVCVQPKEKWVQRYISLL

>itrCcl20
NFDCCLRYTEKVFHHRFLVGFTQQLANEACDIDAVIFYTKRKLAVCADPKKPWVKTAVRRL

>itrCcl25
FEDCCLGYYPKPKLAVLRRASFYRIQEVSGSCNLFAIVFYFRQPGKMVCGNPRDIRVRKAMRLV

>itrCcl28
ASSCCTEVSHHISRRLLERVNSCRIQRADGDCDLAAVILHIKRRRICVSPHNHTIKQWIRAQ

>itrXcl1
KSVCVSLSTQRLPINRIRTYTIKEGSVKAVIFITRRGLKVCANPEAEWVKSAVKSV

Phylogenetic Tree of Rodents, Rabbit and Human Chemokines

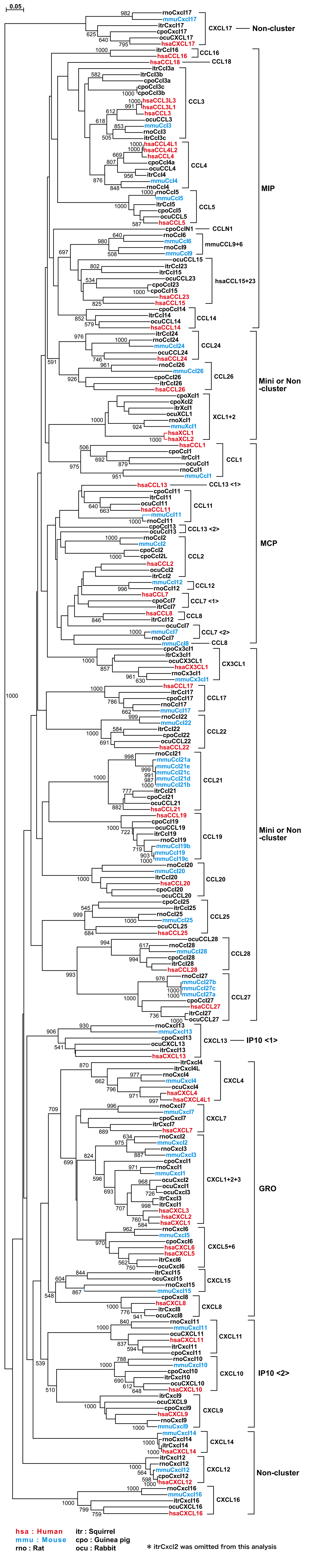

* itrCxcl2 was omitted from this analysis
